# Mechanisms of substrate recognition and *N*^6^-methyladenosine demethylation revealed by crystal structures of ALKBH5–RNA complexes

**DOI:** 10.1093/nar/gkac195

**Published:** 2022-03-25

**Authors:** Simranjeet Kaur, Nok Yin Tam, Michael A McDonough, Christopher J Schofield, Wei Shen Aik

**Affiliations:** Department of Chemistry, Hong Kong Baptist University, Kowloon Tong, Hong Kong SAR, China; Department of Chemistry, Hong Kong Baptist University, Kowloon Tong, Hong Kong SAR, China; The Department of Chemistry and the Ineos Oxford Institute for Antimicrobial Research, Chemistry Research Laboratory, University of Oxford, 12 Mansfield Road, Oxford, OX1 3TA, UK; The Department of Chemistry and the Ineos Oxford Institute for Antimicrobial Research, Chemistry Research Laboratory, University of Oxford, 12 Mansfield Road, Oxford, OX1 3TA, UK; Department of Chemistry, Hong Kong Baptist University, Kowloon Tong, Hong Kong SAR, China

## Abstract

AlkB homologue 5 (ALKBH5) is a ferrous iron and 2-oxoglutarate dependent oxygenase that demethylates RNA *N*^6^-methyladenosine (m^6^A), a post-transcriptional RNA modification with an emerging set of regulatory roles. Along with the fat mass and obesity-associated protein (FTO), ALKBH5 is one of only two identified human m^6^A RNA oxidizing enzymes and is a potential target for cancer treatment. Unlike FTO, ALKBH5 efficiently catalyzes fragmentation of its proposed nascent hemiaminal intermediate to give formaldehyde and a demethylated nucleoside. A detailed analysis of the molecular mechanisms used by ALKBH5 for substrate recognition and m^6^A demethylation is lacking. We report three crystal structures of ALKBH5 in complex with an m^6^A-ssRNA 8-mer substrate and supporting biochemical analyses. Strikingly, the single-stranded RNA substrate binds to the active site of ALKBH5 in a 5′-3′ orientation that is opposite to single-stranded or double-stranded DNA substrates observed for other AlkB subfamily members, including single-stranded DNA bound to FTO. The combined structural and biochemical results provide insight into the preference of ALKBH5 for substrates containing a (A/G)m^6^AC consensus sequence motif. The results support a mechanism involving formation of an m^6^A hemiaminal intermediate, followed by efficient ALKBH5 catalyzed demethylation, enabled by a proton shuttle network involving Lys132 and Tyr139.

## Graphical Abstract

**Graphical Abstract:**
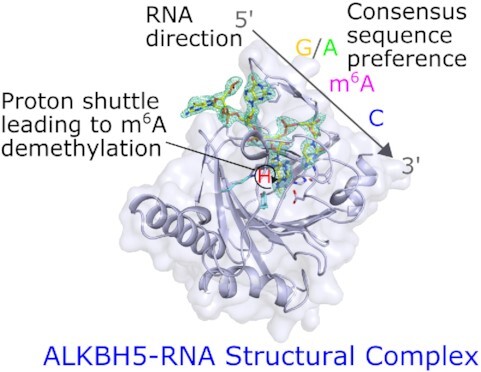
Crystallographic analyses of ALKBH5 bound to a single-stranded m6A-containing RNA substrate reveal details of ALKBH5-substrate interactions and how ALKBH5, unlike the other m6A oxygenase FTO, can catalyze rapid m6A demethylation.

## INTRODUCTION

*N* ^6^-Methyladenosine (m^6^A) is the most abundant stable internal RNA modification and has diverse cellular functions including in regulation of mRNA splicing, RNA processing, nuclear transport, translation and RNA stability (1). ALKBH5 is upregulated in hypoxia and is a hypoxia inducible factor (HIF) target gene (2), suggesting it may play a role in the hypoxic response. Along with the fat mass and obesity-associated protein (FTO), human AlkB homologue 5 (ALKBH5) is one of only two identified human enzymes catalyzing RNA m^6^A methyl group oxidation (3). The m^6^A RNA modification is reported to have important roles in cancer and viral infection (4,5). The m^6^A demethylase activity of ALKBH5 plays roles in the promotion of certain cancers, including glioblastoma, hepatocarcinoma, and acute myeloid leukemia (6–8). ALKBH5 is emerging as a potential drug-target for cancer, however, *ALKBH5* overexpression is reported to inhibit several cancer cell types, including bladder and pancreatic cancers (9,10). Dissecting the apparently conflicting context-dependent roles of m^6^A demethylation in different types of cancer with the use of small molecule probes is of current interest (11).

ALKBH5 is a member of the Fe(II) and 2-oxoglutarate (2OG)-dependent oxygenase (2OG oxygenase) superfamily (2), which catalyzes oxidations of diverse substrates, including nucleic acids, lipids, proteins, and small molecule metabolites (12,13). Within the 2OG oxygenase superfamily, ALKBH5 belongs to the AlkB subfamily, which includes the bacterial *Escherichia coli* nucleic acid repair enzyme AlkB and the human AlkB homologues ALKBH1-8 and FTO.

**Figure 1.**
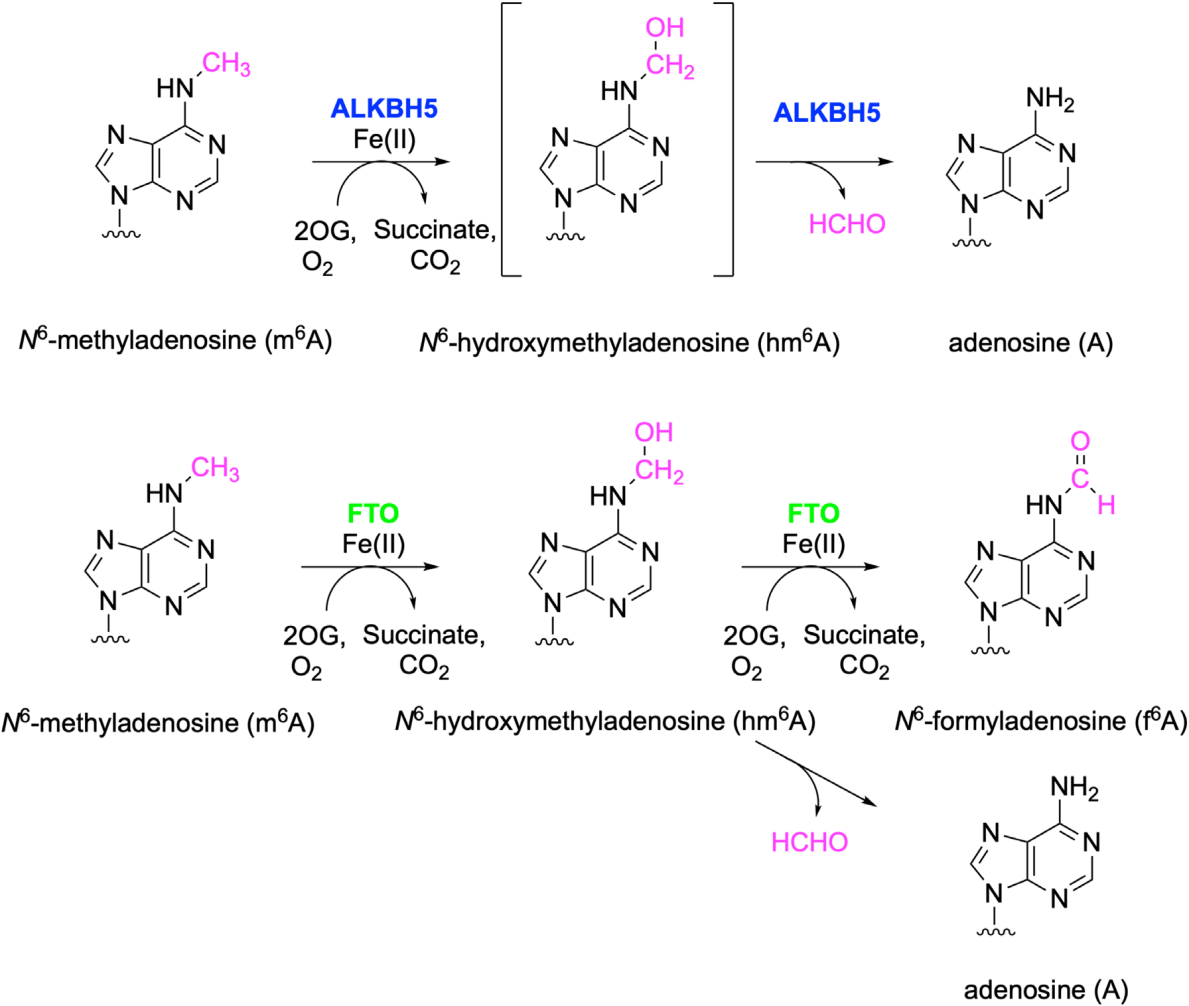
Schematic representation of the ALKBH5 and FTO catalyzed reactions.

ALKBH5 is proposed to demethylate m^6^A in RNA by oxidizing the *N*^6^-methyl group, producing a transient *N*^6^-hydroxymethyladenosine (hm^6^A) intermediate that efficiently fragments to give adenosine and formaldehyde (Figure 1) (12,13). By contrast, enzyme free hm^6^A is a relatively stable species at physiological pH (14,15). The oxidation of m^6^A as catalyzed by FTO is reported to produce a mixture of adenosine, hm^6^A, and *N*^6^-formyladenosine (f^6^A) (Figure 1) (15–17), with hm^6^A being the major product, at least under some assay conditions (14). By contrast with FTO, only the demethylated adenosine product has been observed following ALKBH5 catalysis (17). Recent studies report a role for FTO in specifically demethylating the *N*^6^-methyl of *N*^6^,2′O-dimethyladenosine (m^6^Am) adjacent to the 5′-cap of small nuclear RNA (snRNA) (18). The apparently different substrates and products of FTO and ALKBH5 catalysis may relate to their different physiological roles. Defining detailed structural and mechanistic differences that underlie the different substrate and product selectivities of ALKBH5 and FTO is thus of biological importance with respect to their different roles in RNA regulation and will be useful for the development of selective inhibitors for each.

Structural studies of nucleic acid oxygenases (NAOX) have revealed distinct nucleic acid substrate binding modes. For instance, crystallographic analyses of an FTO variant, AlkB, ALKBH2 and the ten-eleven translocation enzymes (TETs) in complex with their respective ssDNA or dsDNA substrates reveal differences in how they bind their substrates (19–24). We, and others, have determined structures of ALKBH5 in the absence of an RNA substrate (25–28). However, a structure of ALKBH5 bound to an m^6^A-RNA substrate has been unavailable to date. Here, we report crystal structures of ALKBH5 in complex with an m^6^A-containing ssRNA substrate to address this shortfall. The results reveal an unprecedented substrate-binding mode for ALKBH5 relative to other AlkB subfamily oxygenases and enable proposal of a structure-based mechanism rationalizing why, in contrast to FTO, ALKBH5 catalyzes efficient demethylation of m^6^A to A.

## MATERIALS AND METHODS

### Molecular cloning and site-directed mutagenesis

A gene encoding for N-terminally hexahistidine-tagged ALKBH5 residues 74–292 (ALKBH5_74–292_) was cloned into the pNIC28-Bsa4 vector (29) using Transfer-PCR (30). ALKBH5_74–292_ variants were generated by site-directed mutagenesis using Transfer-PCR (30), using the same donor and acceptor plasmid pNIC28-Bsa4-ALKBH5_74–292_ (primers are given in Supplementary Table S2).

### Production and purification of wild type and variant ALKBH5_74–292_ proteins

The plasmid pNIC28-Bsa4-ALKBH5_74–292_ was transformed into *Escherichia coli* Rosetta (DE3) pLysS competent cells and grown in 2YT media supplemented with 50 μg/ml kanamycin and 34 μg/ml chloramphenicol at 37°C. When the cell culture OD_600_ reached 0.8–1.0; expression was induced using a final concentration of 0.8 mM isopropyl β-d-1-thiogalactopyranoside; cells were then grown at 18°C overnight. They were harvested by centrifugation; the cell pellets were resuspended in lysis buffer (25 mM Tris, pH 7.2, 500 mM NaCl, 10 mM imidazole, and 17.4 μg/ml phenylmethanesulfonyl fluoride), and lysed by sonication. The lysates were separated from the insoluble fractions by high-speed centrifugation (Beckman Avanti JA 25.5) at 69673 *g* for 30 min at 4°C. The lysate supernatant was filtered using a syringe filter with pore size of 0.45 μm and purified by nickel affinity chromatography. For structural studies, the lysate supernatant was loaded onto a 5 ml His-trap column (Cytiva, USA) pre-equilibrated with lysis buffer. For biochemical studies, the lysate supernatant was incubated with 0.5 ml Ni-NTA agarose beads (Macherey-Nagel, Germany) at 4°C for 1 h before being loaded onto a gravity column. The columns were washed with 10 column volumes of wash buffer (25 mM Tris, pH 7.2, 500 mM NaCl, 40 mM imidazole) and protein was eluted with 3–4 column volumes of elution buffer (25 mM Tris, pH 7.2, 500 mM NaCl, 375 mM imidazole). The eluate was incubated for 30 min with a final concentration of 100 mM ethylenediaminetetraacetic acid (EDTA) and the His-tag was cleaved by TEV protease (protease to target protein ratio (w/w) of 1:20) overnight while being dialyzed into a buffer containing 25 mM Tris, pH 7.2, 100 mM NaCl and 10 mM β-mercaptoethanol (BME). The eluted proteins were individually purified using a 5 ml HiTrap Heparin column (Cytiva, USA) pre-equilibrated with Buffer A (25 mM Tris, pH 7.2) using a linear gradient of Buffer A and Buffer B (25 mM Tris, pH 7.2, 1 M NaCl). For structural studies, the wild type protein was further purified by size exclusion chromatography using a HiLoad 16/600 Superdex 200 pg column (Cytiva, USA) pre-equilibrated with buffer containing 20 mM MES, pH 6.5, 150 mM NaCl, 1 mM dithiothreitol (DTT). The relevant protein fractions were pooled, concentrated to 47.5 mg/ml, flash frozen in liquid nitrogen and stored at −80°C.

### Production and purification of FTO*Δ*31

FTO*Δ*31 was produced and purified as reported (31). In brief, FTO*Δ*31 was produced in *E. coli* Rosetta pLysS cells and purified by nickel affinity chromatography, treated with a final concentration of 200 mM EDTA, and further purified by heparin affinity, followed by MonoQ ion exchange chromatography. The purified protein was buffered exchanged into 25 mM Tris, pH 7.5, snap frozen in liquid nitrogen and stored at −80°C.

### Crystallization of ALKBH5_74–292_–m^6^A-containing RNA complex

To prepare a near homogeneous ALKBH5_74–292_–ssRNA complex for crystallisation; ALKBH5_74–292_ in 25 mM Tris pH 7.5 buffer, MnCl_2_, *N*-oxalylglycine (NOG), and an 8-mer single-stranded RNA substrate (5′-UGG(m^6^A)CUGC-3′, Horizon Discovery Ltd, UK) were mixed in a 1.2:6:6:1 (ALKBH5_74–292_:MnCl_2_:NOG:RNA) molar ratio in the above order, with a final volume of 1 ml with concentrations of 400 μM ALKBH5_74–292_, 2 mM MnCl_2_, 2 mM NOG and 333 μM RNA substrate. Since NOG was not observed in the active site of the resultant structure, we increased the concentrations of NOG or 2OG 5-fold to increase ligand occupancy. To prepare near homogeneous ALKBH5_74–292_–2OG–ssRNA or ALKBH5_74–292_–NOG–ssRNA for crystallization; ALKBH5_74–292_ in 25 mM Tris, pH 7.5 buffer, MnCl_2_, 2OG or NOG, and an 8-mer single-stranded RNA substrate (5′-UGG(m^6^A)CUGC-3′, Horizon Discovery Ltd, UK) were mixed in a 1.2:6:30:1 (ALKBH5_74–292_:MnCl_2_:2OG/NOG:RNA) molar ratio in the above order, with a final volume of 1 ml with concentrations of 400 μM ALKBH5_74–292_, 2 mM MnCl_2_, 10 mM 2OG or NOG and 333 μM RNA substrate. The mixtures were incubated on ice for 30 min. Each complex was purified by size exclusion chromatography using a Superdex 200 Increase 10/300 GL column (Cytiva, USA), pre-equilibrated with 25 mM Tris pH 7.5 buffer. The fractions corresponding to protein-RNA complex, as determined by UV-vis 260/280 nm ratio (>1), were pooled and concentrated to 17.6 mg/ml (ALKBH5_74–292_–RNA), 11.0 mg/ml (ALKBH5_74–292_–2OG–RNA), and 19.2 mg/ml (ALKBH5_74–292_–NOG–RNA). The protein–RNA complex concentrations were estimated using the summed extinction coefficient (63561 M^−1^ cm^−1^) at 280 nm for both ALKBH5_74–292_ (15930 M^−1^ cm^−1^) and 8-mer m^6^A-containing ssRNA (47631 M^−1^ cm^−1^), which was taken to be the extinction coefficient at 260 nm (74400 M^−1^ cm^−1^) divided by the 260/280 ratio of its UV absorption (1.562).

Crystals of the ALKBH5_74–292_–RNA complex and the ALKBH5_74–292_–NOG–RNA complex were grown at 293 K by the sitting drop vapor diffusion method in CrystalMation Intelli-plate 96-3 High Profile plates (Hampton Research, USA). Crystallization plates were prepared using a Crystal Gryphon dispenser (Art Robbins Instruments, USA). 0.2 μl of 12 mg/ml ALKBH5–RNA complex was mixed with an equal volume of reservoir solution containing 0.2 M sodium sulfate decahydrate, 20% (w/v) polyethylene glycol (PEG) 3350 and equilibrated against 60 μl of reservoir solution. Crystals of the ALKBH5_74–292_–RNA complex grew as rods after two days with typical dimensions 280 μm × 10 μm × 7 μm. 0.2 μl of solution containing final concentrations of 12 mg/ml ALKBH5_74–292_–NOG–RNA complex, 2 mM MnCl_2_ and 10 mM NOG, was mixed with an equal volume of reservoir solution containing 0.2 M sodium formate, 20% (w/v) PEG 3350 and equilibrated against 60 μl of reservoir solution. Crystals of the ALKBH5_74–292_–NOG–RNA complex grew as rods after two days with typical dimensions 170 μm × 15 μm × 7 μm.

To obtain the crystals of the ALKBH5_74–292_–2OG–RNA complex using the hanging drop vapour diffusion method in 24-well VDX plates (Hampton Research, USA) a 1 μl solution containing final concentrations of 10 mg/ml ALKBH5_74–292_–2OG–RNA complex, 2 mM MnCl_2_ and 10 mM 2OG was mixed with an equal volume of reservoir solution containing 0.1 M 2-(*N*-morpholino)ethanesulfonic acid (MES) monohydrate, pH 6.5, 16% w/v PEG 6000 and 5% (v/v) 2-methyl-2,4-pentanediol (MPD); the mixture was equilibrated against 500 μl of reservoir solution at 293 K. Crystals of the ALKBH5_74–292_–2OG–RNA complex grew as rods and appeared after two days with typical dimensions 1000 μm × 50 μm × 40 μm. The reservoir solution was diluted with 100% (v/v) glycerol to a final concentration of 20% (v/v) glycerol for use as cryo-protectant. All crystals were harvested and transferred to the cryo-protectant solution using a nylon loop (Hampton Research, USA), then flash cooled in liquid nitrogen and stored under liquid nitrogen until data collection.

### Data collection and structure determination

X-ray diffraction data were collected from a single crystal of the ALKBH5_74–292_–m^6^A RNA complex at Diamond Light Source Beamline I03 equipped with an Eiger2 XE 16M detector using X-rays tuned to wavelength 0.9763 Å. The autoprocessed data were indexed as high symmetry space group *P*3_1_12, but initial attempts at molecular replacement (MR) failed to provide a reasonable structure solution. Analysis using PHENIX.XTRIAGE (32) indicated merohedral twinning may be present. The data were then re-processed (integrated, merged and scaled) with XIA2 (33) in the lower symmetry space group *P*3_2_. The structure was solved in the *P*3_2_ space group by molecular replacement using PHASER (34) and a structure of ALKBH5_66–292_ (PDB ID 4NJ4) (25) as the search model. Iterative rounds of model building and refinement were performed using COOT (35) and PHENIX.REFINE (32) by including the twin law -*k*, -*h*, -*l* in the refinement parameters until the decreasing *R*_work_ and *R*_free_ no longer converged. X-ray diffraction data for single crystals of either the ALKBH5_74–292_–2OG–m^6^A RNA or ALKBH5_74–292_–NOG–m^6^A RNA complexes were collected at Diamond Light Source Beamline I24 equipped with a Pilatus3 6M detector using X-rays tunned to wavelength 0.9999 Å. The data for the ALKBH5_74–292_–2OG–m^6^A RNA complex were processed (integrated, merged and scaled) using XIA2 in space group *P*3_2_. The structure was solved in the *P*3_2_ space group by molecular replacement using PHASER and the ALKBH5_74–292_–m^6^A RNA complex (PDB ID 7V4G) as the search model. Crystallographic refinement proceeded as above using PHENIX.REFINE by including the twin law -*k*, -*h*, -*l*. For the ALKBH5_74–292_–NOG–m^6^A RNA complex data were collected separately in three sweeps from two rod-shaped crystals (0.1° oscillation per image). Two of three data sets were collected from the same crystal but exposed in different regions so as to avoid radiation damage, the other was collected from a different crystal that was harvested from the same crystallization drop. Autoprocessing using XIA2 indexed each individual data set in the *P*2_1_ space group and twinning was not detected. To improve the completeness and CC$\frac{1}{2}$ value for the high resolution bin, the three datasets were successfully merged. The structure was solved by molecular replacement using PHASER and a structure of the ALKBH5_74–292_-m^6^A RNA complex (PDB ID: 7V4G) as the search model. The model was iteratively fitted and refined using COOT and PHENIX.REFINE, respectively. Crystallographic data processing and refinement statistics can be found in Supplementary Table S1.

### m^6^A demethylation assays

To investigate the activities of ALKBH5_74–292_ and FTO*Δ*31 (FTO residues 32–505) using m^6^A-containing 8-mer RNA substrates containing varied sequences (Horizon Discovery, UK) (Supplementary Table S3), 50 μl reactions containing 0.5 μM ALKBH5_74–292_ or FTO*Δ*31, 300 μM 2OG sodium salt, 2 mM sodium l-ascorbate, 150 μM diammonium iron(II) sulfate, and 4 μM m^6^A-containing 8-mer ssRNA in 25 mM Tris pH 7.5 were performed in triplicate. Reactions were incubated at 37°C for 3 min (ALKBH5_74–292_) or 60 min (FTO*Δ*31) and quenched by adding an equivalent volume of 2 mM EDTA.

To investigate the activities of ALKBH5_74–292_ variants relative to wild type enzyme, 50 μl reactions containing 1 μM ALKBH5_74–292_ (wild type or variants), 300 μM 2OG sodium salt, 2 mM sodium l-ascorbate, 150 μM diammonium iron(II) sulfate, and 8 μM m^6^A-containing ssRNA with the sequence 5′-UGG(m^6^A)CUGC-3′ in 25 mM Tris pH 7.5 were performed in triplicate. The reactions were carried out at 37°C for 7 min and quenched by an equivalent volume of 2 mM EDTA.

The quenched samples (2 μl) were mixed with 1 μl of Matrix Assisted Laser Desorption Ionization (MALDI) matrix (prepared by adding 2 parts of 2,4,6-trihydroxyacetophenone and one part of ammonium citrate) and analyzed using MALDI-Time of Flight (TOF) mass spectrometry (Bruker autoflex^®^ maX) and flexAnalysis software.

## RESULTS AND DISCUSSION

**Figure 2.**
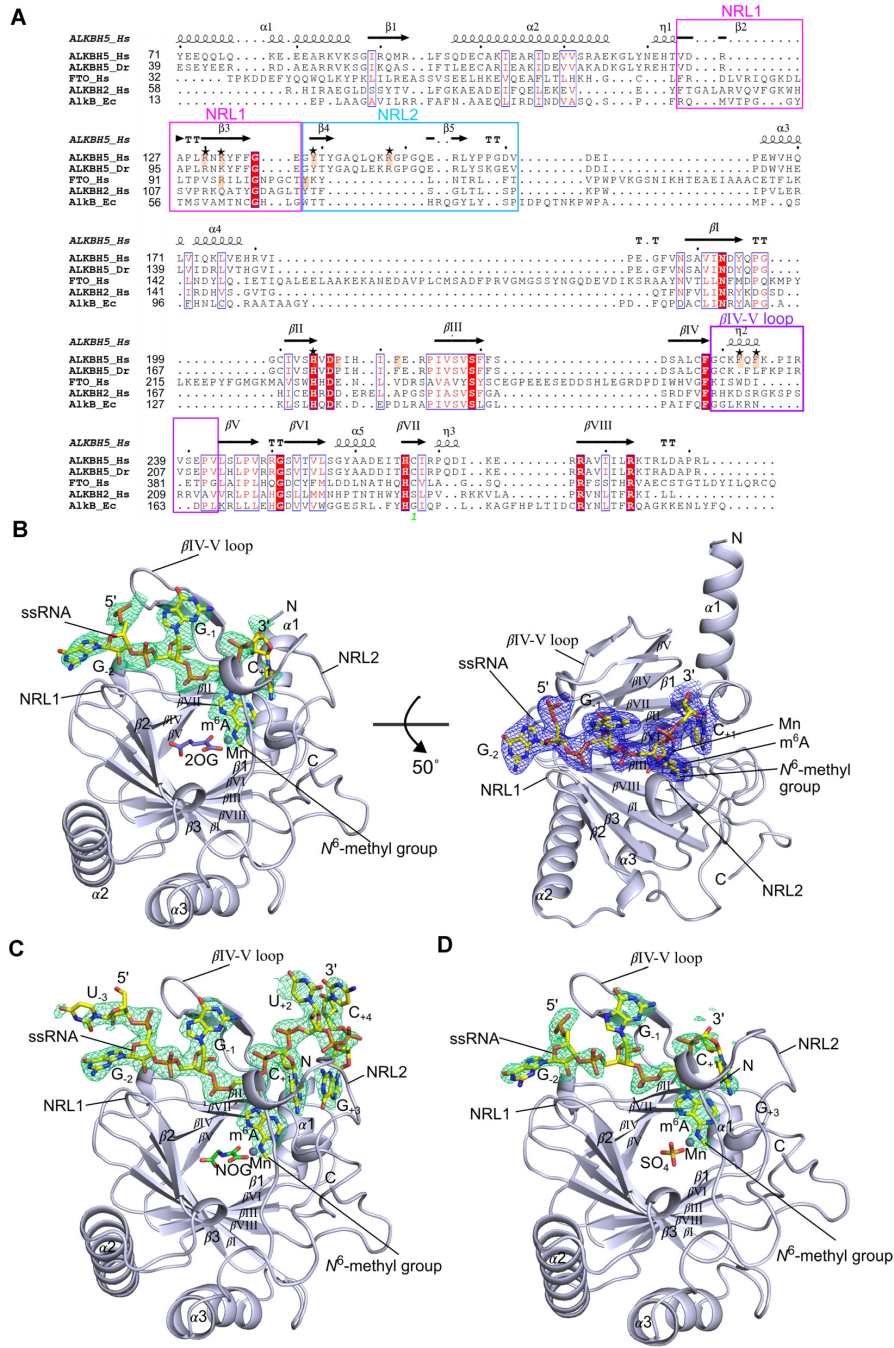
Structures of ALKBH5–ssRNA complexes. (**A**) Structure-based alignment using STRAP (47,48) of representative 2OG oxygenases from the AlkB subfamily. Human ALKBH5 (ALKBH5_Hs) (PDB ID 4NJ4; Uniprot ID Q6P6C2), *Danio rerio* ALKBH5 (ALKBH5_Dr) (PDB ID: 4NPL; Uniprot ID: Q08BA6), human FTO (FTO_Hs) (PDB ID: 3LFM; Uniprot ID: Q9C0B1), human ALKBH2 (ALKBH2_Hs) (PDB ID: 3BTY; Uniprot ID Q6NS38, mismatches: C12S, C110S, G114C, C137S), and *Escherichia coli* AlkB (AlkB_Ec) (PDB ID 2FDJ; Uniprot ID: P05050); secondary structure elements (top) are based on a structure of human ALKBH5 (PDB ID: 4NJ4). Strands of the distorted double-stranded β-helix (DSBH) core are labelled with Roman numerals (*β*I – VIII), *α*-helix (*α*), *β*-strand (*β*), 3_10_ helix (*η*), tight-turn (*TT*), similar residues (blue frames), identical residues (red box and white character), residues with high similarity (red character). The alignment was produced using ESPript 3.0 (https://espript.ibcp.fr) (49). (**B**) Views from the structure of human ALKBH5_74–292_ in complex with 2OG and m^6^A-containing ssRNA (PDB ID: 7WKV). The OMIT electron density map for ssRNA (m*F*_*o*_– D*F_c_*, contoured to 2.5 *σ*) is in green mesh. ssRNA sequence: 5′-UGG(m^6^A)CUGC-3′, only GG(m^6^A)C is observed. Refined electron density map for ssRNA (2m*F*_*o*_– D*F_c_*, contoured to 1.0 *σ*) in blue mesh. (**C**) View from the structure of human ALKBH5_74–292_ in complex with NOG and m^6^A-containing ssRNA (PDB ID: 7WL0). The OMIT electron density map for ssRNA (m*F*_*o*_– D*F_c_*, contoured to 2.5 *σ*) in green mesh. ssRNA sequence: 5′-UGG(m^6^A)CUGC-3′; all eight nucleotides are observed in two of the five complexes in the asymmetric unit; only GG(m^6^A)C is observed in the other three copies. (**D**) Views from the structure of ALKBH5_74–292_ in complex with m^6^A-containing ssRNA and a sulfate ion in the active site (PDB ID: 7V4G). The OMIT electron density map for ssRNA (m*F*_*o*_– D*F_c_*, contoured to 2.5 *σ*) in green mesh. ssRNA sequence: 5′-UGG(m^6^A)CUGC-3′; only GG(m^6^A)C is observed. Note: the m*F*_*o*_– D*F_c_* electron density map is a Sigma-A weighted variant of the *F*_o_– *F*_c_ difference electron density map; the 2m*F*_*o*_– D*F_c_* electron density map is a Sigma-A weighted variant of the 2*F*_o_– *F*_c_ refined electron density map.

In our original structural studies on ALKBH5, in which we reported a structure of ALKBH5_66–292_ (residues 66–292) (25), we attempted co-crystallisation of ALKBH5_66–292_ (residues 66–292) with a 5-mer ssRNA substrate (GGm^6^ACU) by mixing ALKBH5_66–292_, Mn(II), NOG, and 5-mer ssRNA prior to performing crystallization trials; however, this procedure did not lead to crystals. In this study, our initial attempts to prepare a complex of ALKBH5_66–292_–8-mer RNA containing m^6^A using size exclusion chromatography provided clustered crystals unsuitable for diffraction studies. We modified the construct to produce N-terminally truncated ALKBH5_74–292_ (residues 74–292); we were able to co-purify ALKBH5_74–292_ in complex with Mn (substituting for Fe) and the m^6^A-containing 8-mer ssRNA substrate (U_−3_G_−2_G_−1_(m^6^A)_0_C_+1_U_+2_G_+3_C_+4_). Following optimization, crystals were obtained that diffracted to 2.1 Å resolution (Figure 2D) in the *P*3_2_ space group and a structure of ALKBH5_74–292_ in complex with m^6^A-containing RNA was solved by molecular replacement using a structure of human ALKBH5_66–292_ (PDB ID: 4NJ4) (25) as the search model. Three copies of the ALKBH5_74–292_–ssRNA complex are present in the asymmetric unit. Clear electron density is present for four (G_−2_G_−1_(m^6^A)_0_C_+1_) of the eight nucleotides of the ssRNA at the ALKBH5 active site region in all three copies in the asymmetric unit and the observable ssRNA does not appear to be involved in crystal contacts (Supplementary Figure S1). Interestingly, in this initial substrate complex structure the ALKBH5 active site did not contain the cosubstrate 2OG analogue NOG, although NOG had been added to the sample mixture prior to purification by size exclusion chromatography.

Using higher ligand concentrations in the sample mixture and the crystallization drops, we subsequently determined structures of (i) ALKBH5_74–292_ in complex with the same m^6^A-containing 8-mer ssRNA and the 2OG cosubstrate in the *P*3_2_ crystal form to 2.1 Å resolution (Figure 2B), and (ii) a structure of ALKBH5_74–292_ in complex with NOG and the m^6^A-containing 8-mer ssRNA in the *P*2_1_ crystal form to 2.5 Å resolution (Figure 2C). The structure of the ALKBH5_74–292_–NOG–RNA complex in space group *P*2_1_ has five copies of the ALKBH5_74–292_–NOG–ssRNA complex in the asymmetric unit, two of which manifest electron density for all eight nucleotides of the ssRNA substrate (Figure 2C), likely in part due to stabilization by crystal packing contacts with neighboring molecules (Supplementary Figure S2). Four (G_−2_G_−1_(m^6^A)_0_C_+1_) of the eight nucleotides are visible in the other three molecules in the asymmetric unit, in a similar manner for all the ssRNA substrate molecules in both the *P*3_2_ crystal form structures. The three structures from both crystal forms manifest similar overall protein conformations among molecules in the asymmetric unit of each structure, and when compared among the three structures using Chain A from each structure as representatives (RMSDs C*α* 0.2 Å (Chain A from the ALKBH5_74–292_–NOG–RNA complex) and 0.2 Å (Chain A from the ALKBH5_74–292_-RNA complex) relative to Chain A from the ALKBH5_74–292_–2OG–RNA complex). Aside from the four extra nucleotides observed in the *P*2_1_ structure, the active site ssRNA binding modes in all the chains in the asymmetric units of the three structures are similar (Figure 2B–D, Supplementary Figure S3).

To investigate the importance of selected ALKBH5-substrate interactions revealed by the structures and to probe the ALKBH5 mechanisms, we assayed recombinant wild type ALKBH5_74–292_, ALKBH5 active site variants, and FTO*Δ*31 (FTO residues 32–505) using a set of 8-mer m^6^A-containing RNA substrates with varied sequences (Supplementary Table S3) and determined their relative activities using matrix assisted laser desorption ionization mass spectrometry (MALDI-MS) based assays (25) (Supplementary Figure S4). Bases upstream and downstream of the m^6^A were varied individually and in combination in one instance (Supplementary Table S3) to investigate their roles in substrate recognition.

### Overall structure of ALKBH5 in complex with ssRNA

Structures of ALKBH5 in the absence of substrate (25–28) have revealed the 2OG oxygenase superfamily characteristic distorted double-stranded *β*-helix (DSBH) core fold comprising eight β-strands; in the case of ALKBH5, this has an extended βIV–V loop (residues 229–242) containing a 3_10_ helix and has an N-terminal extension, comprised of three α-helices and five β-strands (Figure 2A). Two nucleotide recognition lids (NRL1 (residues 124–137) and NRL2 (residues 138–155)) are common features of the AlkB family of 2OG oxygenases and are positioned within the N-terminal extension of ALKBH5: NRL1 is formed by β-strands 2 and 3 and NRL2 by β-strands 4 and 5. In the reported structures of ALKBH5 (25–28), NRL1 is shorter than in other AlkB subfamily members, while NRL2 is partially disordered.

**Figure 3.**
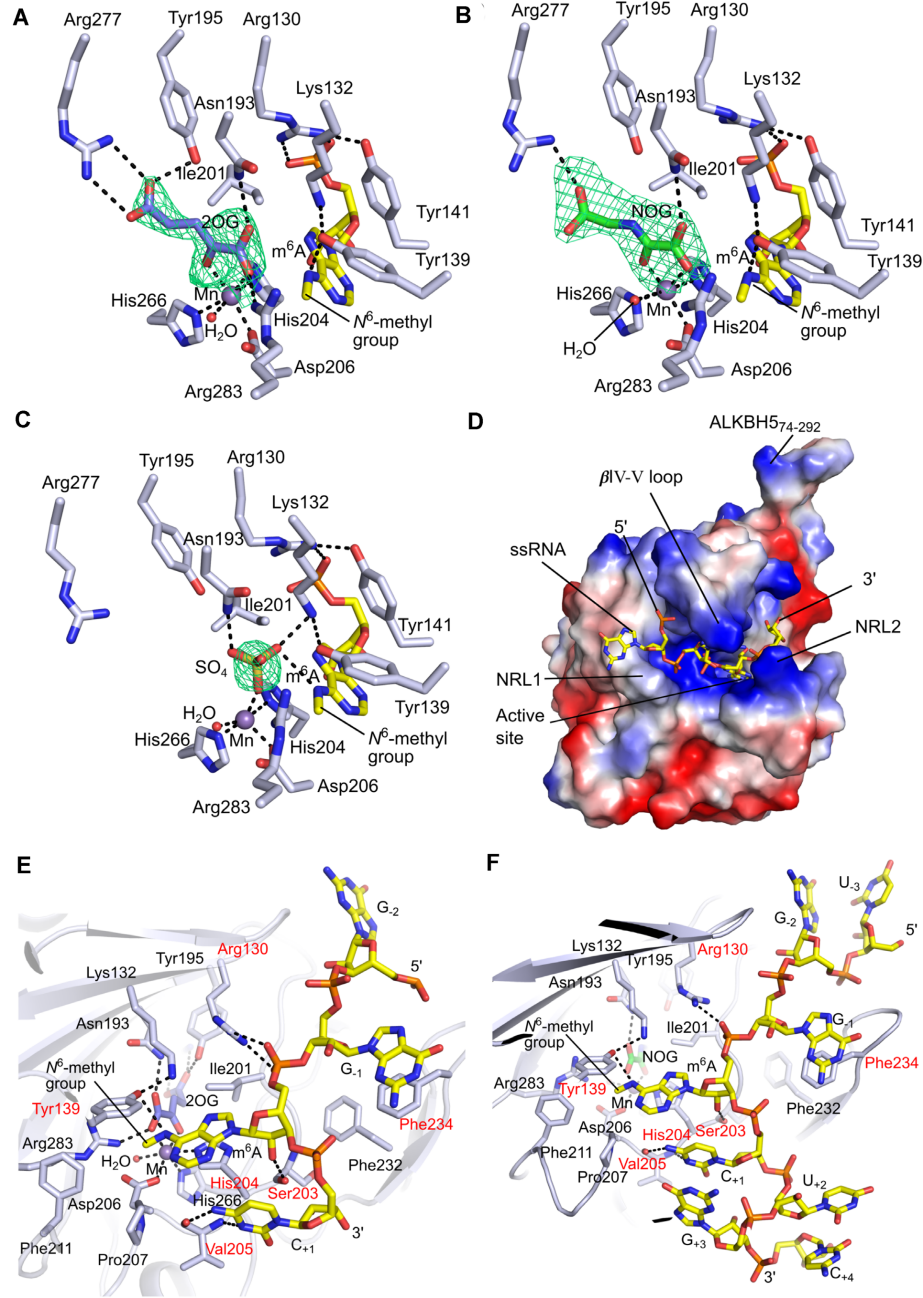
m^6^A ssRNA substrate recognition and 2OG cosubstrate binding by ALKBH5. Views of the active site of ALKBH5_74–292_ with (**A**) 2OG (PDB ID: 7WKV), (**B**), NOG (PDB ID: 7WL0), and (**C**) sulfate ion (PDB ID: 7V4G) bound to the active site metal (Mn substituting for Fe). The OMIT electron density maps for (A) 2OG, (B) NOG and (C) sulfate ion (m*F*_*o*_– D*F_c_*, contoured to 3.0 *σ*) are in green mesh. (**D**) Electrostatic surface potential of the ALKBH5_74-292_–2OG–ssRNA complex (PDB ID: 7WKV) generated using PyMOL (The PyMOL Molecular Graphics System, Version 2.3.0, Schrödinger, LLC). RNA (sticks) is bound into a basic/positively charged cleft (blue). Acidic/negatively charged surface (red), neutral (white). Views of the RNA substrate binding site of structures of (**E**) the ALKBH5_74–292_–2OG–ssRNA complex (PDB ID: 7WKV) and (**F**) the ALKBH5_74–292_–NOG–ssRNA complex (PDB ID: 7WL0). The (A/G)m^6^AC consensus motif is recognized by Phe234, Tyr139, His204 and Val205. RNA-interacting residues are in a red font. Colors: ALKBH5 secondary structure and residues, light blue cartoons/sticks; RNA C, yellow sticks; 2OG C, slate sticks; NOG C, green sticks; O, red; N, blue; P, orange; Mn, purple sphere; water molecule, red sphere; hydrogen bonding/electrostatic interactions, black dashes. Note: m*F*_*o*_– D*F_c_* electron density map is a Sigma-A weighted variant of the *F*_o_– *F*_c_ difference electron density map.

The overall structure of the ALKBH5_74–292_–2OG–ssRNA complex is similar to the human ALKBH5 structures without substrate (25,26,28) (e.g. RMSD C*_α_* = 0.3 Å between Chain A from ALKBH5_74–292_–2OG–RNA complex (PDB ID: 7WKV) and Chain A from ALKBH5_66–292_ without substrate (PDB ID: 4NJ4)), with the exception of conformational changes in the loops (NRL2 and the βIV–V loop) surrounding the active site (Supplementary Figure S5). Clear electron density is apparent for four of the eight ssRNA nucleotides (Figure 2B), which bind in a large positively charged groove extending from the active site (Figure 3D). Compared to the structures without substrate, the βIV–V loop and NRL2 are folded to enclose the substrate at the active site (Figure 3D and Supplementary Figure S5).

At the ALKBH5 active site in the structures reported here, a catalytically inert Mn ion (substituting for catalytically active Fe(II)) is coordinated by the highly conserved metal binding triad His204, Asp206, and His266, a water molecule, and either 2OG, NOG, or a sulfate ion (Figure 3A–C, Supplementary Figure S6A and B). In the structure of the ALKBH5_74–292_–RNA complex without 2OG or NOG, a sulfate ion (likely derived from the crystallization buffer) was observed to ligate to the Mn ion at the 2OG cosubstrate C-1 carboxylate binding site via one of its oxygens; the sulfate ion is also positioned to interact with Asn193 and Lys132 (Figure 3C). The observation of the Mn-ligating sulfate was unexpected because NOG was present in the initial complexation buffer. In the structure of the ALKBH5_74–292_–2OG–RNA complex, the 2OG binding mode is the same as previously reported (PDB IDs: 4OCT and 4NRO) (26,28) (Supplementary Figure S6C). The C-5 carboxylate group of 2OG is positioned to form a salt bridge with the side chain of Arg277. The 2OG ketone oxygen and one of the 2OG C-1 carboxylate oxygens chelate to the metal in a bidentate manner and form a hydrogen bond and electrostatic interactions with the side chains of Asn193 and Arg283, respectively (Figure 3A and Supplementary Figure S7). In the structure of the ALKBH5_74–292_–NOG–RNA complex, the NOG adopts a highly similar binding mode to 2OG, although with slight variations in the conformation of its C-5 carboxylate group (Figures 3B and Supplementary Figure S8).

Recent NMR studies of the ALKBH5 2OG binding mode to an active site Zn(II) ion, coupled with molecular dynamics (MD) studies suggest a mixture of ‘off-line’ and ‘on-line’ 2OG C-1 carboxylate binding modes (36). The crystallographically observed 2OG and NOG binding modes in ALKBH5 when complexed with Mn and an m^6^A substrate are of the ‘off-line’ mode. However, our crystallographic data may represent a snapshot of the complex in the catalytic process, where rearrangement of the 2OG C-1 carboxylate could occur. Another difference between our crystallographic studies and the reported molecular dynamics studies (36) is that the modeled binding mode of the ssDNA substrate to ALKBH5 in the MD simulations was based on a structure of the ALKBH2–dsDNA complex (PDB ID: 3RZJ) (37), and thus the 5′ to 3′ direction of the modeled nucleic acid is reversed relative to that of the ssRNA substrate when bound to ALKBH5 as observed in our crystallographic studies (see later).

### Conformational changes of ALKBH5 upon substrate binding

Comparison of the ALKBH5 structures with and without ssRNA reveals major conformational changes, implying induced fit upon substrate binding. The conformational changes primarily occur in the NRL2 and the βIV–V loop bordering the active site (Supplementary Figure S5). In contrast with structures of ALKBH5 without substrate, where NRL2 is partially disordered (25–28), when a substrate is bound the entire NRL2 loop is well ordered. NRL2 residues interact with the ssRNA substrate on the 3′ side of the m^6^A. The β-hairpin of NRL2 observed in the absence of substrate (25) is distorted upon substrate binding, forming a 3_10_ helix followed by random coil (Figure 2B and Supplementary Figure S5). The βIV–V loop makes contact with the RNA substrate oligomer on the 5′ side of the m^6^A; the 3_10_ helix observed in the βIV–V loop without substrate is transformed to a β-hairpin on substrate binding (Figures 2B and Supplementary Figure S5) (25,26,28). Secondary structure changes of NRL2 and the βIV–V loop as well as rigidification of NRL2 was also observed in the recently reported NMR studies by Purslow *et al.* (36), consistent with our crystallographic observations. The m^6^A-containing ssRNA binds across the active site opening and is enclosed by NRL1, NRL2 and the βIV–V loop (Figure 2B and Supplementary Figure S5). Our structural analyses support the reported chemical shift perturbations by NMR for residues on NRL1, NRL2 and the βIV–V loop (36). These three structural elements are shaped like ‘two-fingers and a thumb’, that collectively grip the ssRNA. The conformational changes resulting in the substrate being near entirely enclosed at the active site region are reminiscent of analogous changes observed on substrate binding with other 2OG dependent oxygenases, i.e. human HIF prolyl hydroxylase domain 2 (PHD2), *Pseudomonas* PHD (PPHD), *Trichoplax adhaerens* PHD (TaPHD), and human *γ*-butyrobetaine hydroxylase 1 (BBOX1) (38–41). Electrostatic surface potential analysis of ALKBH5_74–292_ shows that most of its substrate-binding cleft is positively charged, so enabling ionic interactions with negatively charged oligonucleotide substrates (Figure 3D).

### ALKBH5 prefers m^6^A consensus motif (A/G)m^6^AC

**Figure 4.**
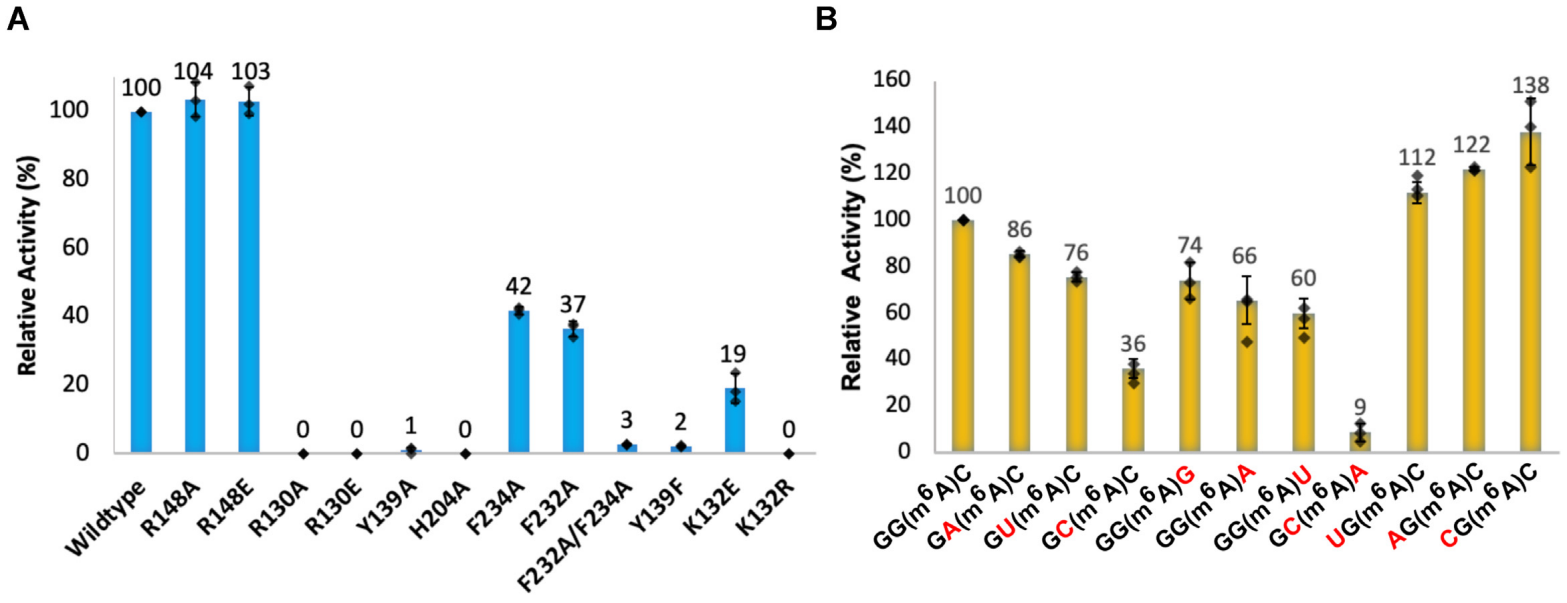
Activities of wild type and variant ALKBH5_74–292_ and with different ssRNA substrates. (**A**) Relative activities of wild type ALKBH5_74–292_ and variants of ALKBH5_74–292_. (**B**) Relative activities of wild type ALKBH5_74–292_ with m^6^A-containing 8-mer ssRNA sequence variants at positions –2, –1 and +1; m^6^A is at position 0. Nucleotides that are varied from original sequence used in crystallographic studies (…G_−2_G_−1_(m^6^A)_0_C_+1_…) are in red. Heights of the bar graphs indicate the mean (*n* = 3); black diamond markers indicate individual data points; error bars indicate standard deviation of the mean (*n* = 3).

To explore how ALKBH5 selectively binds its proposed (A/G)m^6^AC substrate consensus RNA motif (42,43), we systematically investigated interactions between each observed nucleotide base and the protein in the ALKBH5-substrate structural complexes. The m^6^A inserts into the active site with the 5′-phosphate of (m^6^A)_0_ interacting with Arg130 from NRL1 (average distance 3.0 Å) (Figures 3E and F). The side chain of Arg148 is generally disordered, but in a number of protein molecules in the ALKBH5_74-292_–2OG–RNA (Chains A and C), ALKBH5_74-292_–NOG–RNA (Chain C) and ALKBH5_74-292_–RNA (Chains A, B and C) complexes, the observed side chain electron density for Arg148 suggests that it may interact with the C_+1_ 5′-phosphate group (average distance 3.3 Å) in solution (Supplementary Figure S9). The 2′-OH group of the m^6^A ribose forms a hydrogen bond with the main chain carbonyl of Ser203 (average distance 2.8 Å) (Figure 3E and F). The m^6^A nucleobase likely forms a *π*-*π* interaction with the imidazole ring of His204, and the *N*^6^-amine is positioned within hydrogen bonding distance to the side chain OH of Tyr139 (average distance 3.2 Å) although the geometry in the crystal structure is not ideal for strong hydrogen bonding; we cannot rule out small adjustments of this interaction during catalysis in solution (Figure 3E and F). The *N*^6^-methyl group of m^6^A is positioned towards a hydrophobic region formed by the side chains of Phe211, Tyr139, and, to a lesser extent, Pro207; these residues also project towards the Watson–Crick edge of the m^6^A base (Figure 3E and F). This arrangement appears to help position the m^6^A methyl group close to the metal ion (Figure 3E and F). The R130A, R130E and H204A variants were inactive while the Y139A variant had little or no activity (∼1% of wild type) (Figure 4A), validating the proposals based on structural observations that these are key residues in substrate binding and/or catalysis. However, the NRL2 loop variants R148A and R148E show similar activities to the wild type (Figure 4A), implying the potential interaction between the Arg148 side chain and the C_+1_ phosphoryl group is not critical.

G_−1_ is positioned ∼180° about the phosphoribosyl backbone relative to m^6^A, and one face of its six-membered ring is positioned to make *π*-*π* interactions with the side chain of Phe234 on the βIV–V loop (Figure 3E and F). This observation supports the proposal that ALKBH5 selectively binds the larger bicyclic purine nucleobases (G or A) over smaller monocyclic pyrimidines (C or U) at its –1 binding position, potentially due to more effective π–π overlap with the Phe234 side chain. To test this hypothesis, we investigated the activities of purified ALKBH5_74–292_ with different sequence substitutions (either A, C or U) at position –1. The results show that ALKBH5_74–292_ has higher activity with G_−1_ (100%) and A_−1_ (86 ± 1%) compared to U_−1_ (76 ± 2%) and C_−1_ (36 ± 4%) (Figure 4B), in support of the proposed preference for purines at the -1 position. The F234A and F232A variants both have reduced, but substantial activity (42 ± 1 and 37 ± 2%, respectively of wild type). However, the double F232A/F234A variant manifests severely impaired activity (<3% of wild type) (Figure 4A). Thus, F234 is important in substrate recognition while F232 may also play a role in substrate recognition.

The cytosine base at the +1 position is adjacent to the m^6^A nucleobase at the active site opening. Importantly, a pair of hydrogen bonds are formed between the cytosine N3 and *N*^4^-amine atoms and the main chain amide NH and carbonyl O atoms of Val205, respectively (average distances 3.1 and 3.0 Å, respectively) (Figure 3E and F). The interactions that C_+1_ makes with ALKBH5 imply that a bulkier purine base (A or G) at this position might require alteration of the RNA backbone conformation due to steric hindrance with the main chain at Val205. A uracil base at the +1 position will result in the loss of a hydrogen bond, because the 4-carbonyl group of uracil is unable to form a hydrogen bond with the main chain carbonyl of Val205, so explaining why a cytosine is preferred at the +1 position (consistent with the (A/G)m^6^AC consensus motif for ALKBH5) (42,43). Indeed, our *in vitro* activity assays show that ALKBH5 has reduced activity with RNA sequences with G, A or U at the +1 position (74 ± 8%, 66 ± 10% and 60 ± 7% respectively) relative to sequences containing C at the +1 position (100%) (Figure 4B), in accord with structural observations suggesting a preference for the (A/G)m^6^AC consensus motif by ALKBH5. Strikingly, when we tested the activity of an 8-mer ssRNA substrate containing both a pyrimidine (C_−1_) at –1 position and a purine (A_+1_) at +1 position (…C_−1_(m^6^A)_0_A_+1_…), the activity of ALKBH5 was severely reduced (9 ± 4%) (Figure 4B), supporting the proposal that the identity of the nucleobases directly adjacent to m^6^A can have a dramatic effect on activity.

Recognition of the G nucleobase at the –2 position appears to be less selective, as it does not form direct interactions with ALKBH5 (Supplementary Figure S10), consistent with a m^6^A consensus motif (A/G/U)(A/G)m^6^AC(42, 43). We tested ALKBH5 activity using RNA substrate sequences with U, A or C at the –2 position; these sequences all resulted in higher activity (112 ± 4%, 122 ± 1% and 138 ± 14%, respectively) than the substrate with a G at –2. Analysis of the protein structures does not provide an obvious rationale for these higher activities with nucleobases other than G at the –2 position, which might in part reflect intramolecular ssRNA interactions. Further studies employing modeling are of interest to explore this observation.

Overall, our structural and biochemical data suggest that ALKBH5 prefers substrate sequences with the (A/G)m^6^AC motif, consistent with the prevalence of m^6^A in biological contexts, including DRACH motifs, where A is the potentially N6-methylated adenosine (D is A, G or U; R is A or G; and H is A, C or U) (42,43). In cells there is evidence that DRACH motifs are substrates for the RNA m^6^A methyltransferases METLL3/METTL14 complex and subsequently ALKBH5 (44). Our work supports the proposed action of ALKBH5 on DRACH motifs. Interestingly, however, with FTO no discernible consensus sequence preference for the tested substrates was identified (Supplementary Figure S11). Although further detailed *in vitro* and *in vivo* studies are required, these observations suggest that ALKBH5 and FTO might target m^6^A in different sequence (or RNA secondary structure) contexts, consistent with their different biological roles.

### The RNA phosphoribosyl backbone binding direction is reversed in ALKBH5 compared to substrates of several AlkB subfamily members

To further investigate how ALKBH5 confers substrate selectivity, we compared the ALKBH5-ssRNA structures with the available structures of nucleic acid oxygenases (NAOX) in complex with their substrates. The results reveal a striking difference in the 5′-3′ phosphoribosyl backbone binding direction for ALKBH5 compared to other NAOX substrate structural complexes, *i.e*. that the phosphoribosyl backbone of the ssRNA binds to ALKBH5 in an opposite direction to that observed in structures of: (i) an FTO variant in complex with ssDNA (PDB ID: 5ZMD); (ii) ALKBH2 in complex with dsDNA (PDB ID: 3BTY); (iii) AlkB in complex with ssDNA or dsDNA (PDB IDs: 2FD8 and 3BIE) (Supplementary Figures S12 and S13) (21–23). Furthermore, the facial orientation of the m^6^A nucleobase in ALKBH5 is opposite to that observed in FTO (Supplementary Figure S13), an observation that likely impacts on catalysis and substrate selectivity/type of product (see below).

### Demethylation vs hydroxylation reaction mechanisms

**Figure 5.**
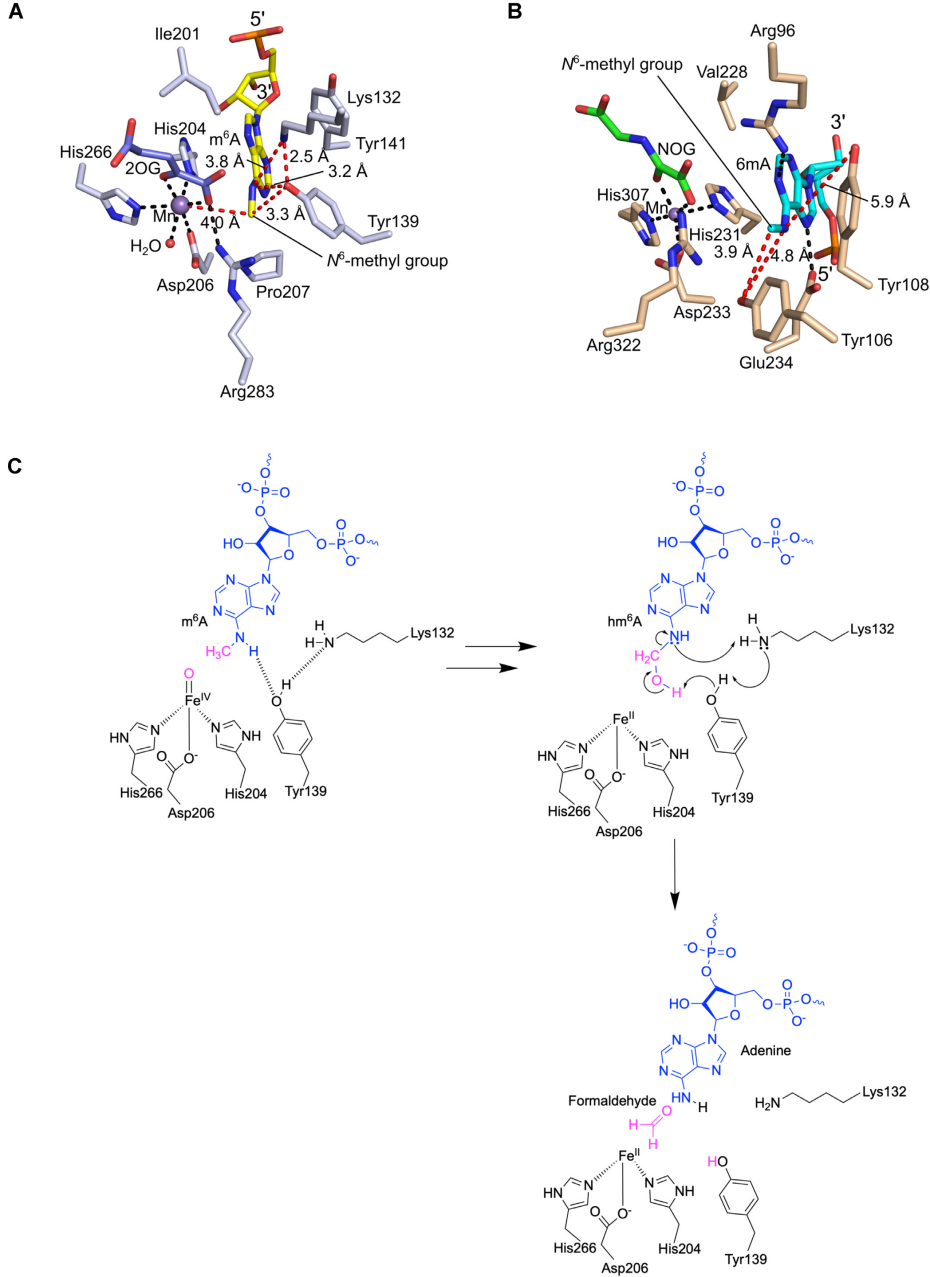
Proposed role for a proton shuttle in ALKBH5-catalyzed m^6^A hydroxylation followed by demethylation. (**A**) View of the active site of ALKBH5_74-292_–2OG–m^6^A RNA complex (PDB ID: 7WKV; Chain A). Colors: ALKBH5 residue C (light blue sticks), m^6^A C (yellow), 2OG C (slate), O (red), N (blue), P (orange), Mn (purple sphere), water molecule (red sphere), interactions (black dashes), distances (red dashes). (**B**) View of the active site of FTO variant-6mA ssDNA complex (PDB ID: 5ZMD) (22). Colors: FTO residue C (light brown), 6mA C (cyan), NOG C (green), O (red), N (blue), P (orange), Mn (purple sphere), interactions (black dashes), distances (red dashes). Note: the binding orientation of the face of 6mA nucleotide is inverted in FTO relative to that of m^6^A in ALKBH5 reflecting their opposing 5′ to 3′ nucleotide directions. (**C**) Proposed outline mechanism for demethylation of m^6^A catalyzed by ALKBH5. Note the proposed roles of Lys132 and Tyr139 in ALKBH5 catalysed m^6^A demethylation (17) and that for FTO directly analogous residues appear to be absent explaining why it does not (efficiently) catalyze demethylation.

We next focused on comparing the ALKBH5 and FTO active sites as both oxidize m^6^A in a manner ultimately leading to demethylation, though recent studies have shown that FTO generates hm^6^A as its major nascent product, at least in the tested sequence contexts (14,17). We were particularly interested in investigating the potential consequences on demethylation of the inverted 5′ to 3′ ssRNA substrate direction through the ALKBH5 active site cleft compared with that of FTO. In the case of FTO, in addition to a structure with a *N*^3^-methylthymidine nucleoside in the active site (45), a structure of an FTO variant with *N*^6^-methyldeoxyadenosine (6mA) bearing ssDNA is reported (Figures 5A and B, and Supplementary Figure S9) (22). Wild type FTO and the variant used for structural studies have been shown to be active against 6mA in ssDNA, although the available evidence is that mRNA is likely to be a more physiologically relevant substrate (16,22).

In the ALKBH5_74–292_-2OG-substrate complex, the *N*^6^-methyl group of m^6^A is positioned ∼4.0 Å away from the Mn (Figure 5A), a substrate-metal distance consistent with a (near) catalytically productive orientation as observed in other structures of 2OG oxygenase enzyme-substrate complexes (46). The phenolic hydroxyl of Tyr139 is positioned to form a hydrogen bond with N6 of m^6^A, and with Lys132. As proposed (17), the proximity of N^ϵ^ of Lys132 N to Tyr139 OH (average distance: 2.9 Å) and the m^6^A N6 (average distance 3.9 Å), and Tyr139 OH to the m^6^A *N*^6^-methyl (average distance: 3.3 Å) enables efficient demethylation following initial hydroxylation to give hm^6^A (Figure 5C). Upon hydroxylation of m^6^A, the side chain hydroxyl group of Tyr139 may participate in a proton shuttle by donating a proton to the side chain amine of Lys132 while receiving a proton from the hydroxyl group of hm^6^A. The deprotonated hm^6^A can then undergo fragmentation forming formaldehyde and an adenosine. During fragmentation, the N6 of adenosine accepts a proton from the protonated amine of Lys132, completing the proton transfer cycle. This mechanism allows m^6^A to be enzymatically hydroxylated and demethylated in quick succession, i.e. it accounts for the lack of hm^6^A detected in ALKBH5 assays.

Substitutions of the ALKBH5 active site residues Lys132 and Tyr139 were carried out to investigate their roles in hm^6^A fragmentation. Our results support the importance of these residues in overall catalysis as identified by Toh *et al.* (17). The K132R (to mimic the arginine present in FTO (Arg96_FTO_) – see below) and Y139A ALKBH5 variants were inactive and the K132E and Y139F variants manifested only 19 ± 4% and 2 ± 0% demethylation activities relative to wild type ALKBH5 (Figure 4A).

Comparison of the ALKBH5-ssRNA complex structure with that of an FTO variant in complex with *N*^6^-methyldeoxyadenosine (6mA) in ssDNA implies that residues in the active site of FTO are not positioned to participate in a proton shuttle in an analogous manner to that we propose for ALKBH5 (Figure 5B). While in FTO there are two tyrosine residues (Tyr106 and Tyr108) near the 6mA base, these residues are significantly farther away from the N6 of 6mA (4.8 Å for Tyr106 OH and 5.9 Å for Tyr108 OH) compared to the Tyr139 OH to N6 distance (average distance: 3.2 Å) in ALKBH5. Importantly, the inverted orientation of the face of the m^6^A nucleobase in ALKBH5 compared to FTO allows Arg96_FTO_, which occupies a similar position as Lys132 of ALKBH5, to form a hydrogen bond with N1 of m^6^A (3.0 Å), thus its ability to supply a proton to N6 of m^6^A during potential fragmentation to give formaldehyde is less likely (the arginine guanidino group is also less likely to act as a base due its potentially higher intrinsic pKa compared to the N^ϵ^-amino group of lysine). Consequently, FTO-catalyzed m^6^A oxidation produces hm^6^A-containing RNA as a major product, which is a relatively stable species at physiological pH (14,15,17).

## CONCLUSIONS

Three crystal structures of ALKBH5 in complex with its ssRNA substrate and co-substrate/co-substrate mimic reveal an unprecedented substrate binding mode for a 2OG oxygenase. Comparison of FTO and ALKBH5 substrate structural complexes provides a structural basis for the different type of products made by each of the two enzymes with m^6^A substrates, i.e. demethylation with formaldehyde formation (ALKBH5) or *N*-methyl hydroxylation (FTO). This knowledge should be useful in the development of selective ALKBH5 and FTO inhibitors, including those exploiting the different substrate binding modes of the two oxygenases. The results further illustrate the remarkably flexible nature of substrate (nucleic acid) binding by and reactivity of 2OG oxygenases, despite the presence of a conserved DSBH fold harboring closely related Fe(II) and 2OG binding elements. The results also provide structural information on why ALKBH5 prefers substrates with m^6^A located within the consensus (A/G)m^6^AC motif.

## DATA AVAILABILITY

Atomic coordinates and structure factors for the reported crystal structures have been deposited with the Protein Data Bank under accession numbers 7V4G, 7WKV, and 7WL0.

## Supplementary Material

gkac195_Supplemental_File
