# Supplementary material for: Mechanisms of substrate recognition and N6-methyladenosine demethylation revealed by crystal structures of ALKBH5–RNA complexes
Source: Nucleic Acids Res. 2022 Mar 25;50(7):4148–60. doi: 10.1093/nar/gkac195 (PMC9023255; doi:10.1093/nar/gkac195)
Supplement: gkac195_Supplemental_File [file gkac195_supplemental_file.pdf]

Mechanisms of Substrate Recognition and N^6-Methyladenosine Demethylation Revealed by Crystal Structures of ALKBH5-RNA Complexes

Simranjeet Kaur[1], Nok Yin Tam[1], Michael A. McDonough[2,*], Christopher J. Schofield[2,*],

Wei Shen Aik[1,*]

[1] Department of Chemistry, Hong Kong Baptist University, Kowloon Tong, Hong Kong SAR, China.
[2] The Department of Chemistry and the Ineos Oxford Institute for Antimicrobial Research, Chemistry Research Laboratory, University of Oxford, 12 Mansfield Road, Oxford, OX1 3TA, United Kingdom.

To whom correspondence should be addressed. Tel: +852 3411 6682; Fax: +852 3411 7063; Email: aikweishen@hkbu.edu.hk. Correspondence may also be addressed to Michael A. McDonough. Tel: +44 01865 275 629; Fax: +44 01865 285 002; Email: michael.mcdonough@chem.ox.ac.uk; and Christopher J. Schofield. Tel: +44 01865 275 625; Fax: +44 01865 285 002; Email: christopher.schofield@chem.ox.ac.uk

SUPPLEMENTARY DATA

TABLE OF CONTENTS

Figures S1 to S13

Tables S1 to S3

Supplementary References

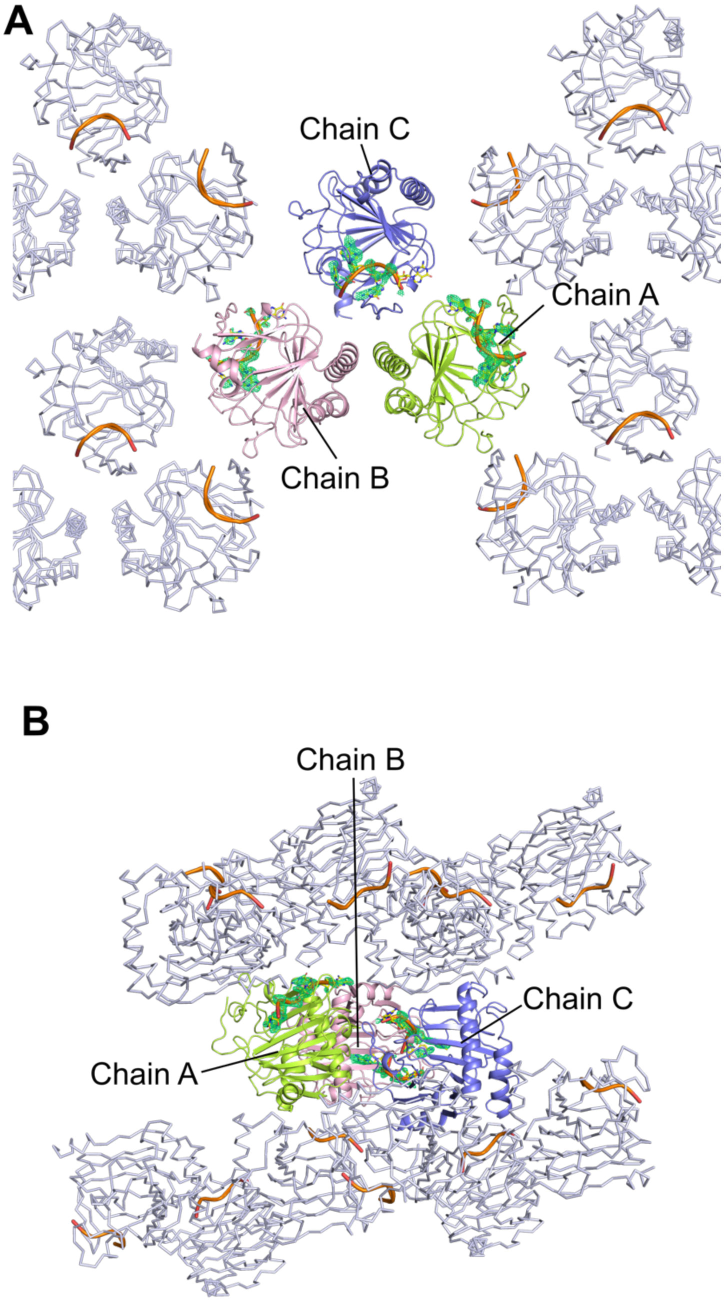

Figure S1. Crystal packing of the ALKBH5$_{74\text{-}292}$-m^{6}A ssRNA complex (PDB ID 7V4G). OMIT electron density maps for ssRNA (mF$_o$-DF$_c$, contoured to 2.5 σ) are in green mesh, the RNA backbone is in orange or yellow sticks, ALKBH5$_{74\text{-}292}$ Chain A is a lemon green cartoon, Chain B is a pink cartoon, and Chain C is a deep blue cartoon. There are three copies of the ALKBH5$_{74\text{-}292}$-ssRNA complex in each asymmetric unit.

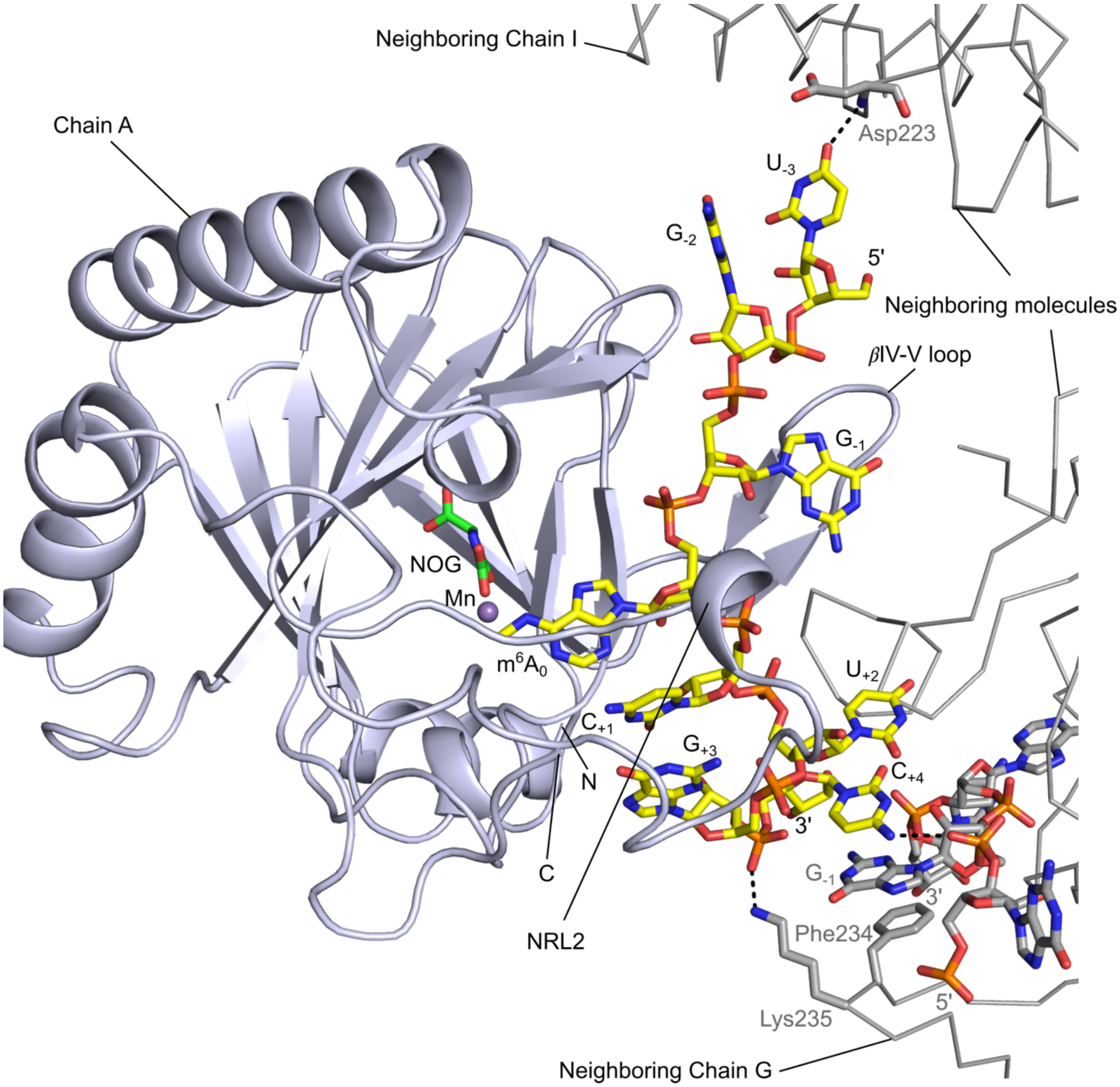

Figure S2. Crystal packing stabilization of terminal ssRNA nucleobases in the ALKBH5$_{74\text{-}292}$-NOG-RNA structure (PDB ID 7WL0). Residues from a symmetry related neighboring complex involved in crystal packing stabilization are in sticks. Note that U_{+2}, C_{+4}, the 'neighboring' G_{-1} and the 'neighboring' side chain of Phe234 form multiple layers of π-π stacking interactions; Lys235 from a neighboring molecule interacts with the 5´-phosphate of G_{+3}. Colors: ALKBH5$_{74\text{-}292}$, light blue cartoon; RNA, yellow sticks; neighboring molecules, grey ribbon and sticks; NOG, green sticks; Mn, purple sphere; O, red; N, blue; P, orange; hydrogen bonding/electrostatic interactions, black dashes.

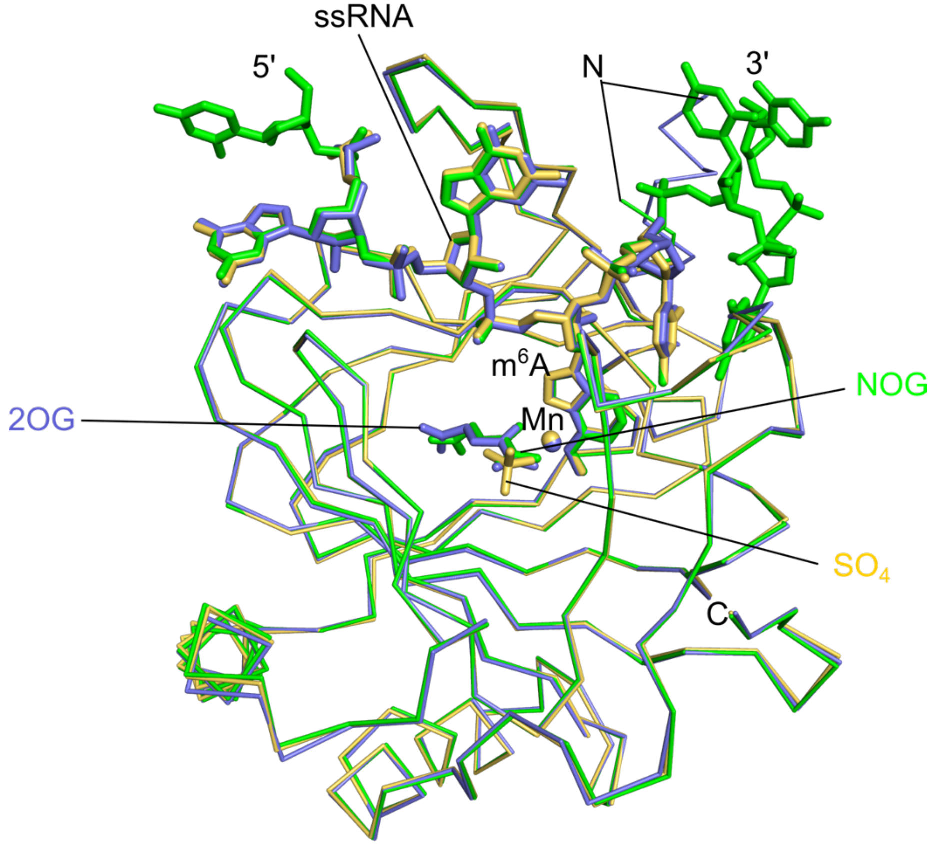

Figure S3. Superimposition of structures of the ALKBH5$_{74\text{-}292}$-2OG-RNA Chain A (slate) (PDB ID 7WKV), ALKBH5$_{74\text{-}292}$-NOG-RNA Chain A (green) (PDB ID 7WL0), and ALKBH5$_{74\text{-}292}$-RNA Chain A (yellow) (PDB ID 7V4G) complexes. Note: the ssRNAs in all three structures have similar binding modes to ALKBH5 and additional nucleotides are observed in Chains B and F (not shown) of the $P2_1$ crystal form of ALKBH5$_{74\text{-}292}$-NOG-RNA (PDB ID 7WL0).

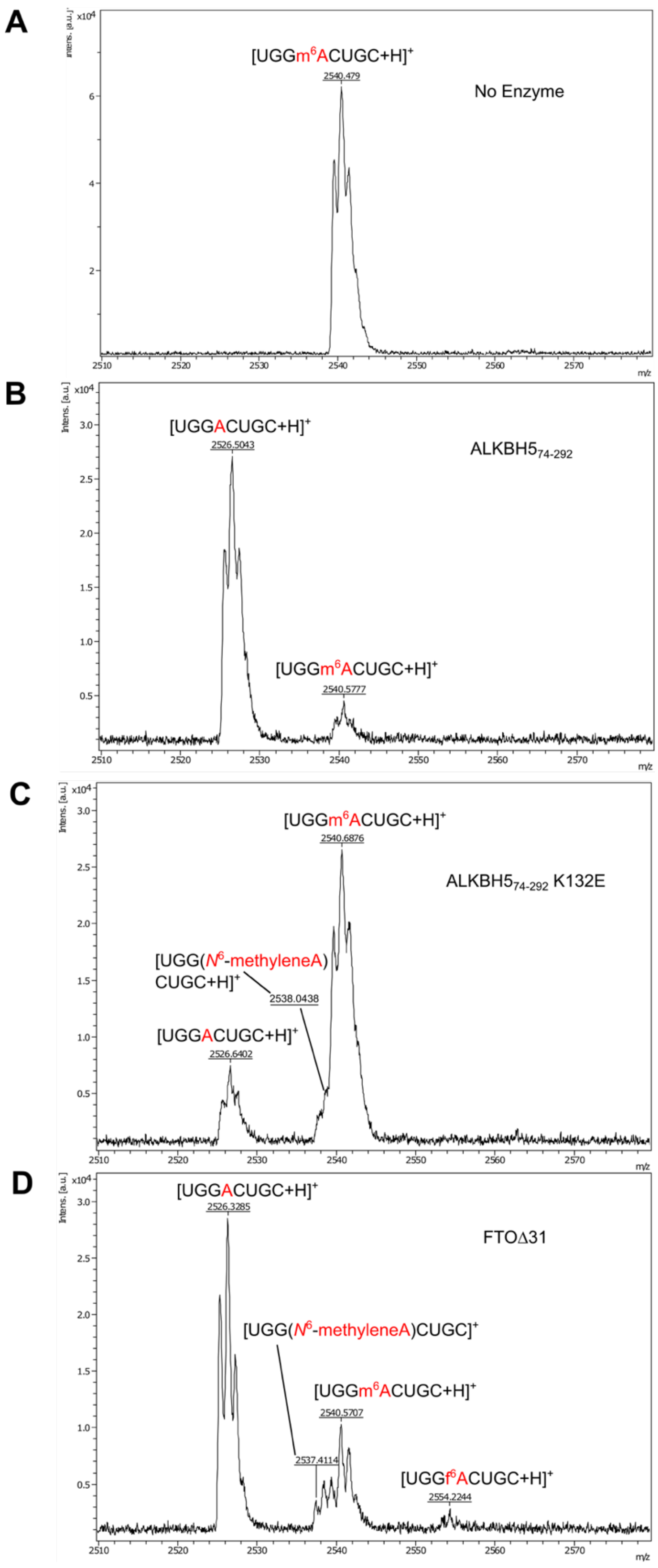

Figure S4. Representative MALDI-TOF mass spectra from demethylation assays with purified ALKBH5$_{74-292}$. (A) No enzyme control, (B) wild type ALKBH5$_{74-292}$, (C) ALKBH5$_{74-292}$ K132E, and (D) FTO*Δ*31. Molecular weight of RNA substrate: 2539.6 Da. In the case of the K132E variant (C), in addition to adenosine, a small peak with a mass of -2 Da relative to the substrate mass is observed corresponding to N^6-methyleneadenosine, as also observed in our MALDI-TOF analysis of reactions catalyzed by FTO*Δ*31 (D) and in a previous report (1).

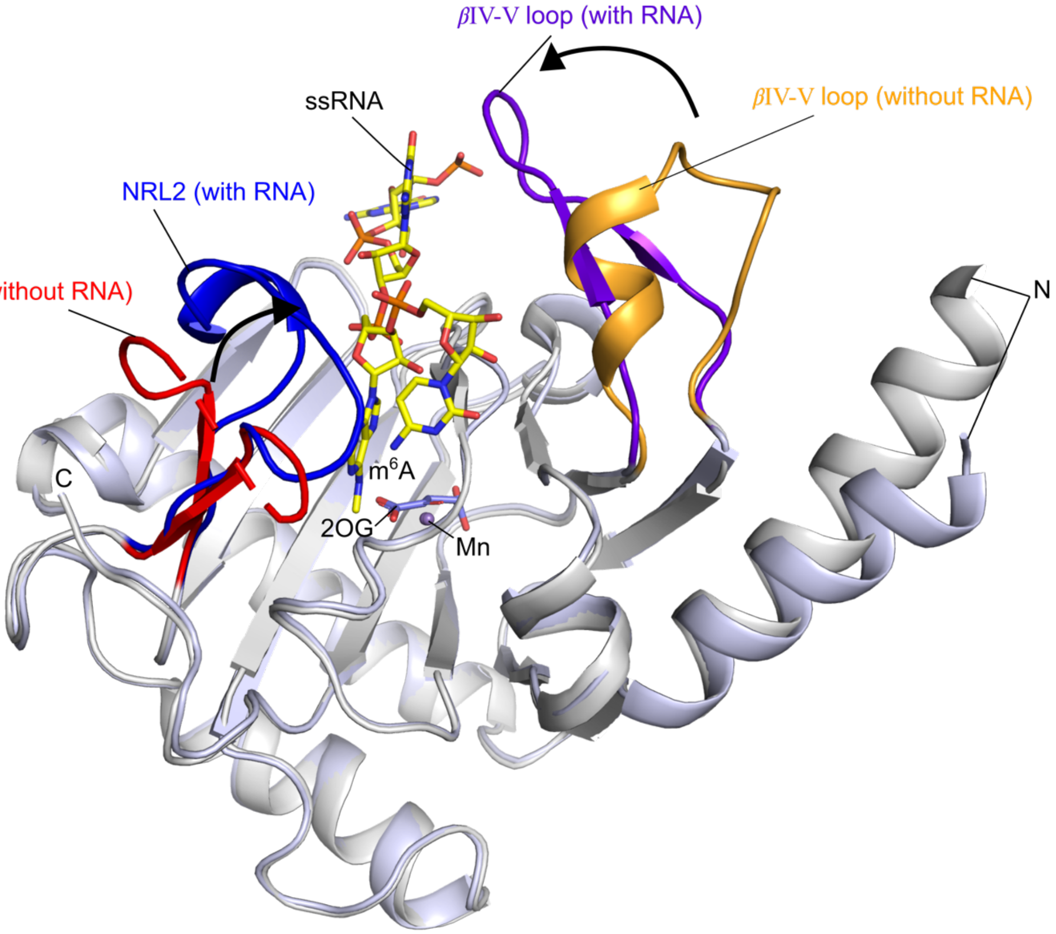

Figure S5. A view of the conformational changes observed in the ALKBH5 loops NRL2 and βIV-V upon ssRNA substrate binding. Superimposition of Chain A from the structure of the ALKBH5$_{74\text{-}292}$-2OG-ssRNA complex (light blue cartoon) (PDB ID 7WKV) with Chain A from a structure of ALKBH5 without substrate (white cartoon) (PDB ID 4NJ4) (2). Note that the N-terminal helix of the structure of ALKBH5 without substrate (PDB 4NJ4) is of a longer construct (residues 66-292), thus a longer N-terminal helix is observed. Colors: NRL2 in the absence of RNA, red cartoon; NRL2 with RNA bound (yellow sticks), blue cartoon; βIV-V loop in the absence of RNA, orange cartoon; βIV-V loop with RNA bound, purple cartoon.

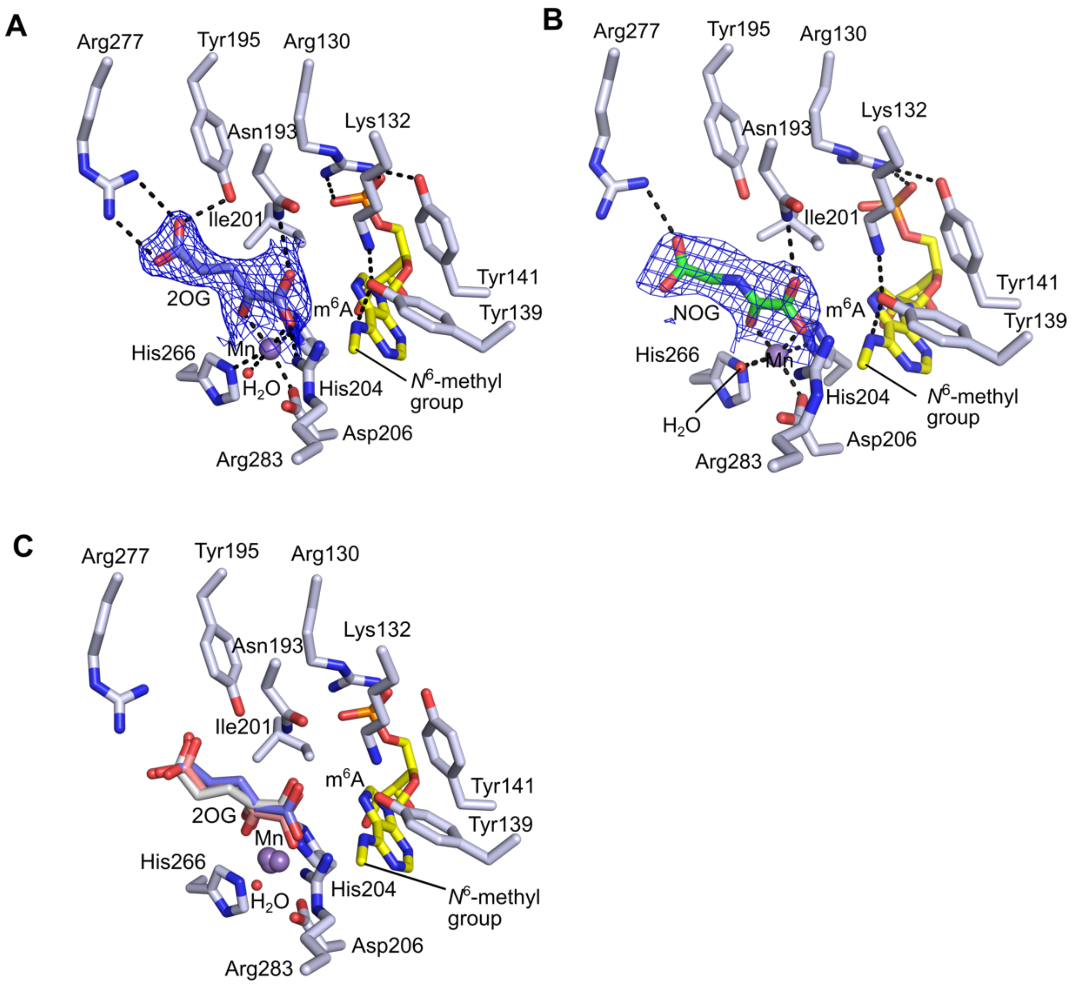

Figure S6. ALKBH5 2OG binding site. Views of the active site of ALKBH5$_{74\text{-}292}$ with (A) 2OG (PDB ID 7WKV), and (B) NOG (PDB ID 7WL0) bound to the active site metal (Mn substituting for Fe). The refined electron density maps for (A) 2OG and (B) NOG (2mF$_o$-DF$_c$, contoured to 1.0 σ) are in blue mesh. (C) A superimposition of Chain A from the structure of the ALKBH5$_{74\text{-}292}$-2OG-ssRNA complex (PDB ID 7WKV) with Chain A from a structure of ALKBH5 in complex with 2OG, but without RNA (PDB ID 4OCT) (3) and another structure of ALKBH5 in complex with 2OG only (PDB ID 4NRO) show a similar 2OG binding mode regardless of the presence or absence of oligonucleotide substrate. Key 2OG binding site residues from the structure of ALKBH5$_{74\text{-}292}$-2OG-RNA complex (PDB ID 7WKV) are shown in sticks. Colors: for the ALKBH5$_{74\text{-}292}$-2OG-RNA complex (PDB ID 7WKV) and ALKBH5$_{74\text{-}292}$-NOG-RNA complex (PDB ID 7WL0), ALKBH5$_{74\text{-}292}$, light blue; 2OG, slate; for the ALKBH5-2OG complex (PDB ID 4OCT) (3), 2OG, salmon; for another ALKBH5-2OG complex (PDB ID 4NRO) (4), 2OG, white; NOG C, green sticks; RNA, yellow; O, red; N, blue; P, orange; Mn, purple sphere; water molecule, red sphere; hydrogen bonding/electrostatic interactions, black dashes. Note: 2mF$_o$-DF$_c$ electron density map is a Sigma-A weighted variant of the 2F$_o$-F$_c$ refined electron density map.

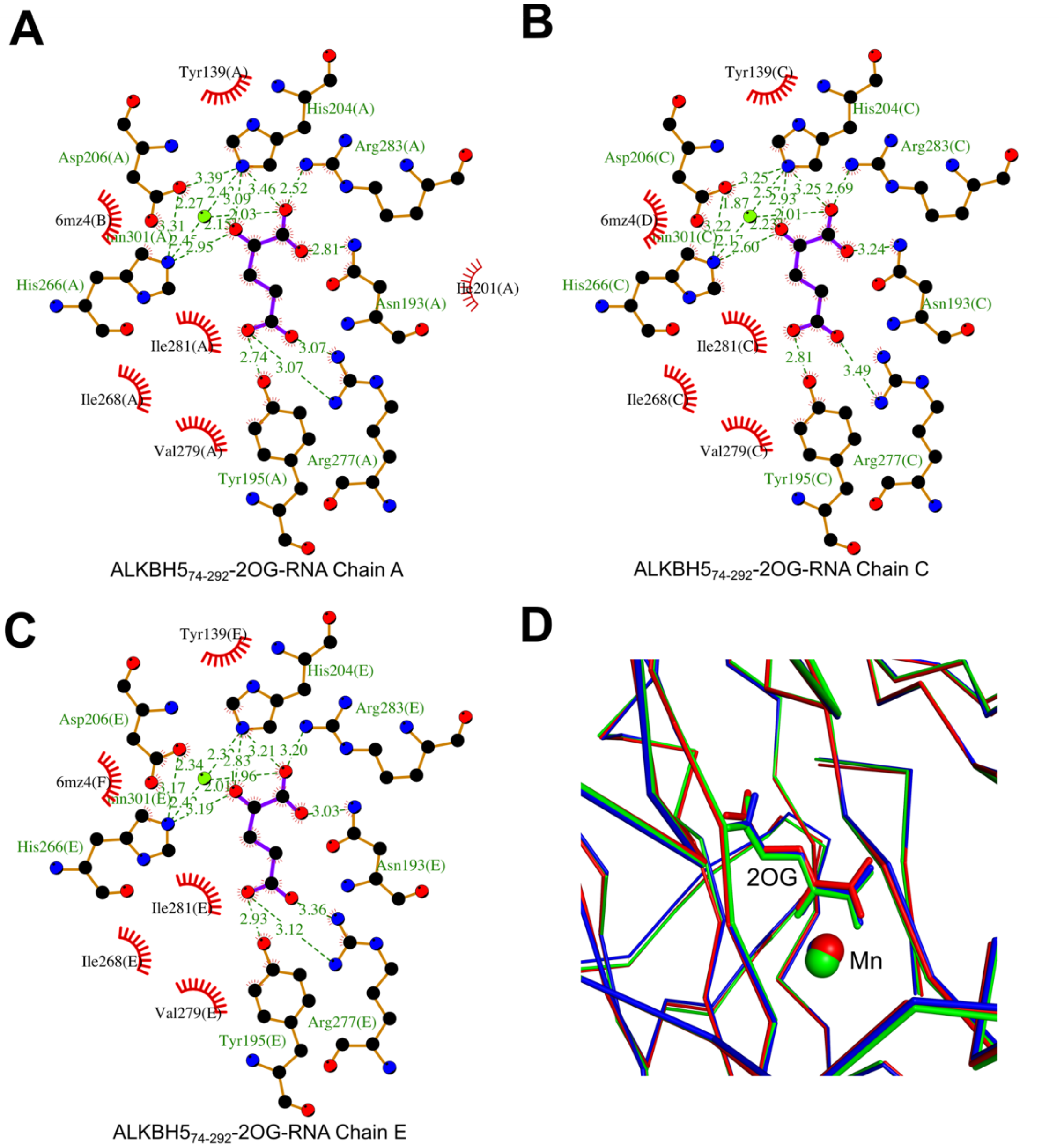

Figure S7. Binding modes of 2OG in various chains from the structure of the ALKBH5$_{74\text{-}292}$-2OG-RNA complex (PDB ID 7WKV). 2-Dimensional plots representing interactions of 2OG with residues within the active sites of (A) Chain A, (B) Chain C, and (C) Chain E in the structure of the ALKBH5$_{74\text{-}292}$-2OG-RNA complex (PDB ID 7WKV) generated by Ligplot+ (5, 6). Hydrogen bonds are indicated by green dashes; hydrophobic interactions, red arcs. (D) View of 2OG (sticks) binding modes in a 3-dimensional overlay of all 3 chains in the asymmetric unit from the structure of the ALKBH5$_{74\text{-}292}$-2OG-RNA complex (PDB ID 7WKV). Colors: Chain A, red; Chain C, green; Chain E, blue.

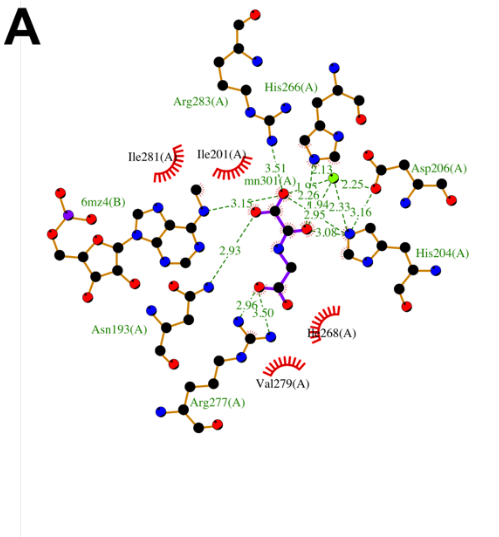

ALKBH5$_{74\text{-}292}$-NOG-RNA Chain A

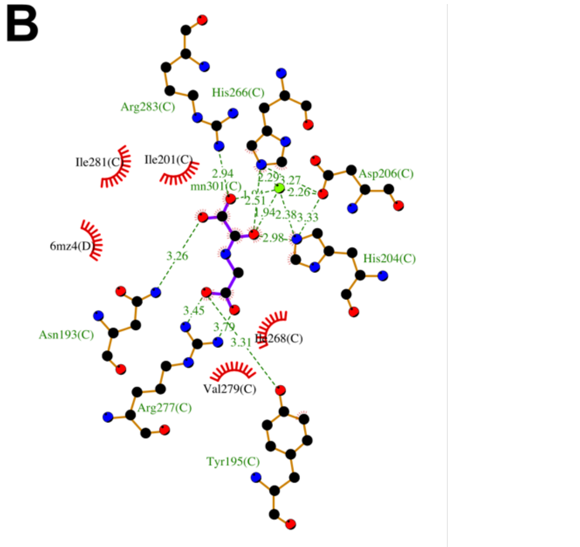

ALKBH5$_{74\text{-}292}$-NOG-RNA Chain C

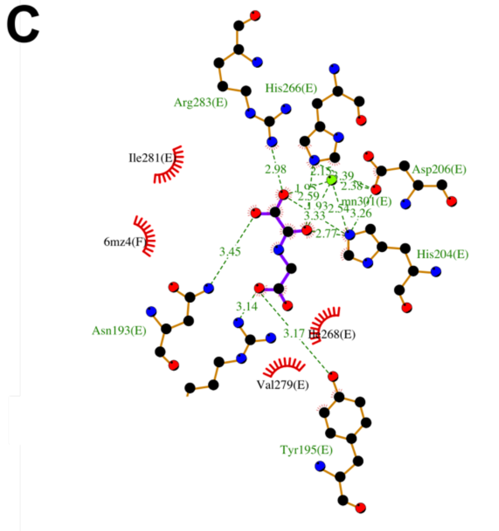

ALKBH5$_{74\text{-}292}$-NOG-RNA Chain E

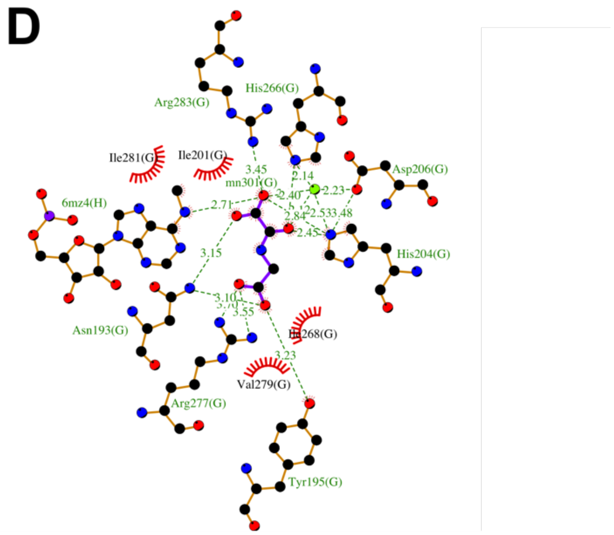

ALKBH5$_{74\text{-}292}$-NOG-RNA Chain G

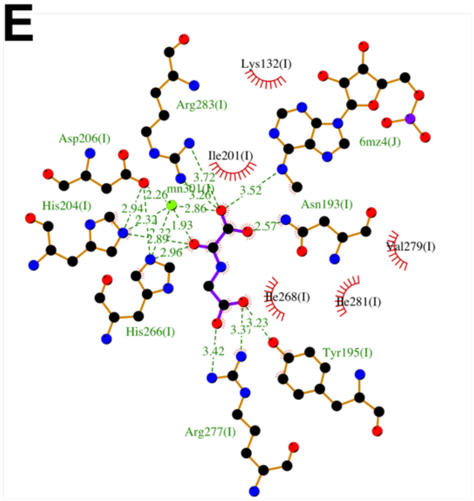

ALKBH5$_{74\text{-}292}$-NOG-RNA Chain I

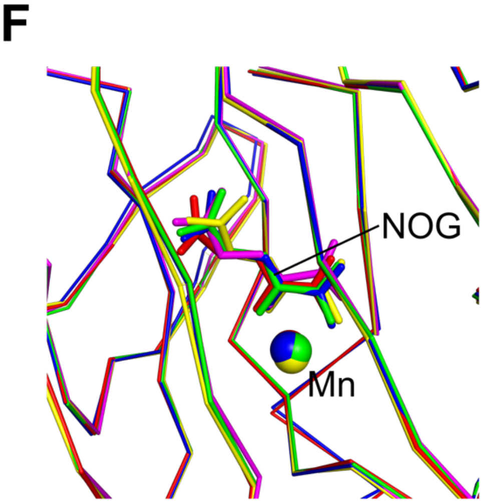

Figure S8. Binding modes of NOG in various chains from the structure of the ALKBH5$_{74\text{-}292}$-NOG-RNA complex (PDB ID 7WL0). 2-Dimensional plots representing interactions of NOG with residues within the active sites of (A) Chain A, (B), Chain C, (C), Chain E, (D) Chain G, and (E) Chain I in the structure of the ALKBH5$_{74\text{-}292}$-NOG-RNA complex (PDB ID 7WL0) generated by Ligplot+ (5, 6). Hydrogen bonds are indicated by green dashes; hydrophobic interactions, red arcs. (F) View of NOG (sticks) binding modes in a 3-dimensional overlay of all 5 chains in the asymmetric unit from the structure of the ALKBH5$_{74\text{-}292}$-NOG-RNA complex (PDB ID 7WL0). Colors: Chain A, red; Chain C, green; Chain E, blue; Chain G, yellow; Chain I, magenta.

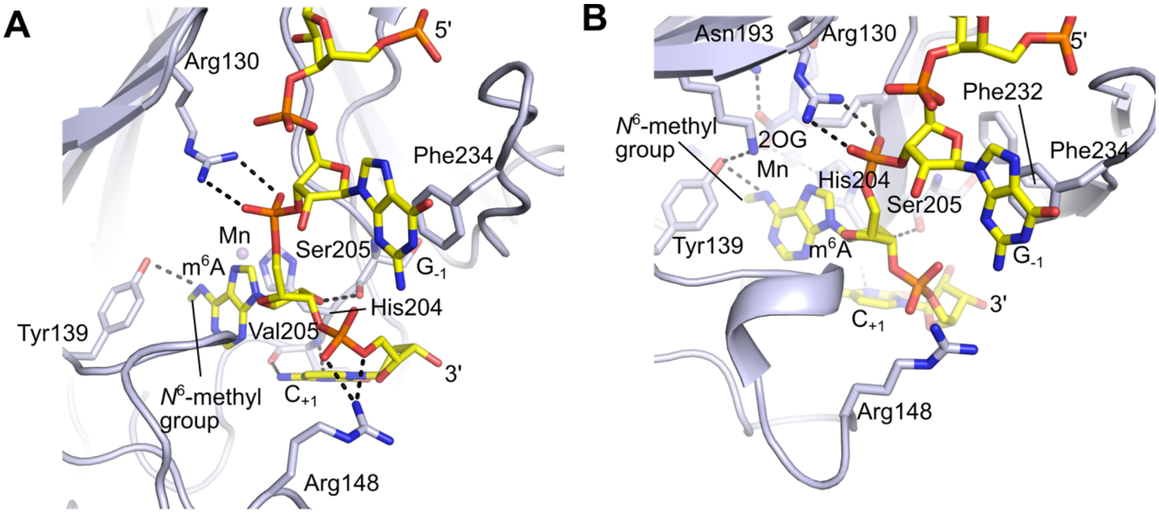

Figure S9. Potential interaction of Arg148 with C_{+1}. Views of the RNA binding sites from the structures of (A) the $ALKBH5_{74\text{-}292}$-RNA complex (PDB ID 7V4G) Chain A and (B) the $ALKBH5_{74\text{-}292}$-2OG-RNA complex (PDB ID 7WKV) Chain A. Note: the side chain of Arg148 is likely predominantly disordered (Chain E of $ALKBH5_{74\text{-}292}$-2OG-RNA (PDB ID 7WKV) and Chains A, E, G and I of $ALKBH5_{74\text{-}292}$-NOG-RNA (PDB ID 7WL0)). Colors: ALKBH5 secondary structure represented as light blue cartoon and residues are shown as sticks; RNA C, yellow sticks; 2OG C, slate sticks; O, red; N, blue; P, orange; Mn, purple sphere; water molecule, red sphere; hydrogen bonding/electrostatic interactions, black dashes.

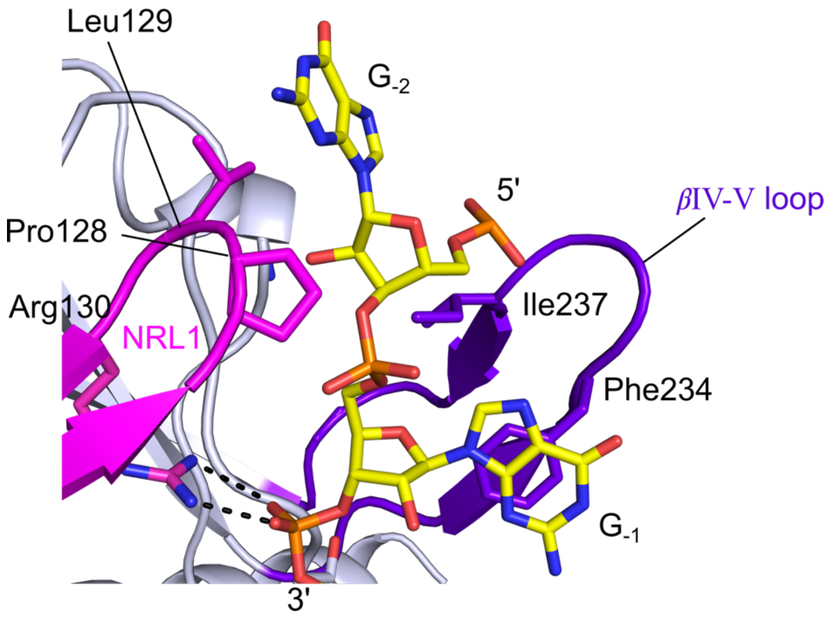

Figure S10. View of the ALKBH5$_{74\text{-}292}$ structural elements surrounding the G$_{-2}$ nucleotide of the bound substrate. This image is from Chain A of the structure of the ALKBH5$_{74\text{-}292}$-2OG-ssRNA complex (PDB ID 7WKV). Note: G$_{-2}$ is mostly surrounded by solvent, and does not appear to make specific interactions with protein. Colors: ALKBH5$_{74\text{-}292}$, light blue cartoon; NRL1, magenta; βIV-V loop, purple; RNA, yellow sticks; O, red; N, blue; P, orange. Hydrogen bonds or electrostatic interactions are indicated by black dashes.

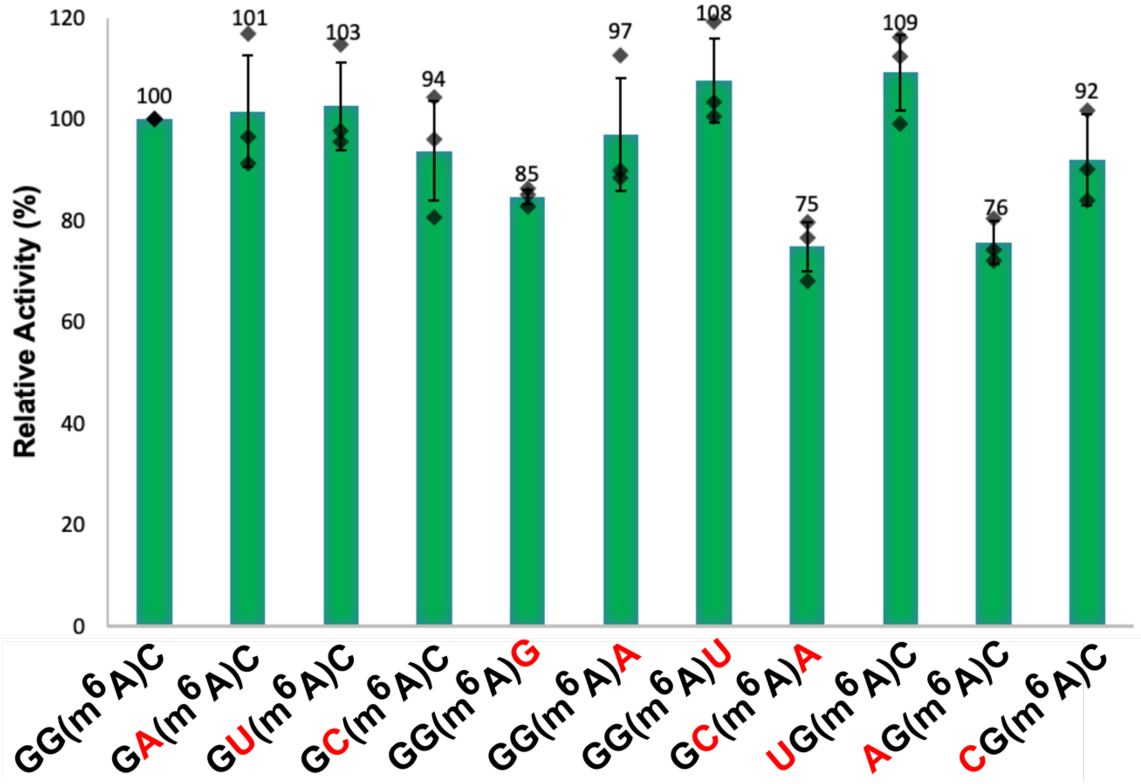

Figure S11. Relative activities of the N-terminally truncated FTOΔ31 enzyme with m^{6}A-containing an 8-mer ssRNA sequence UGG(m^{6}A)CUGC (m^{6}A at position 0) varied at positions -2, -1, and +1. Nucleotides that are varied from the original UGG(m^{6}A)CUGC sequence used in crystallographic studies (…$G_{-2}G_{-1}(m^6A)_0C_{+1}$…) are in red. Heights of the bar graphs indicate the mean (n = 3); black diamond markers indicate individual data points; error bars indicate standard deviation of the mean (n = 3).

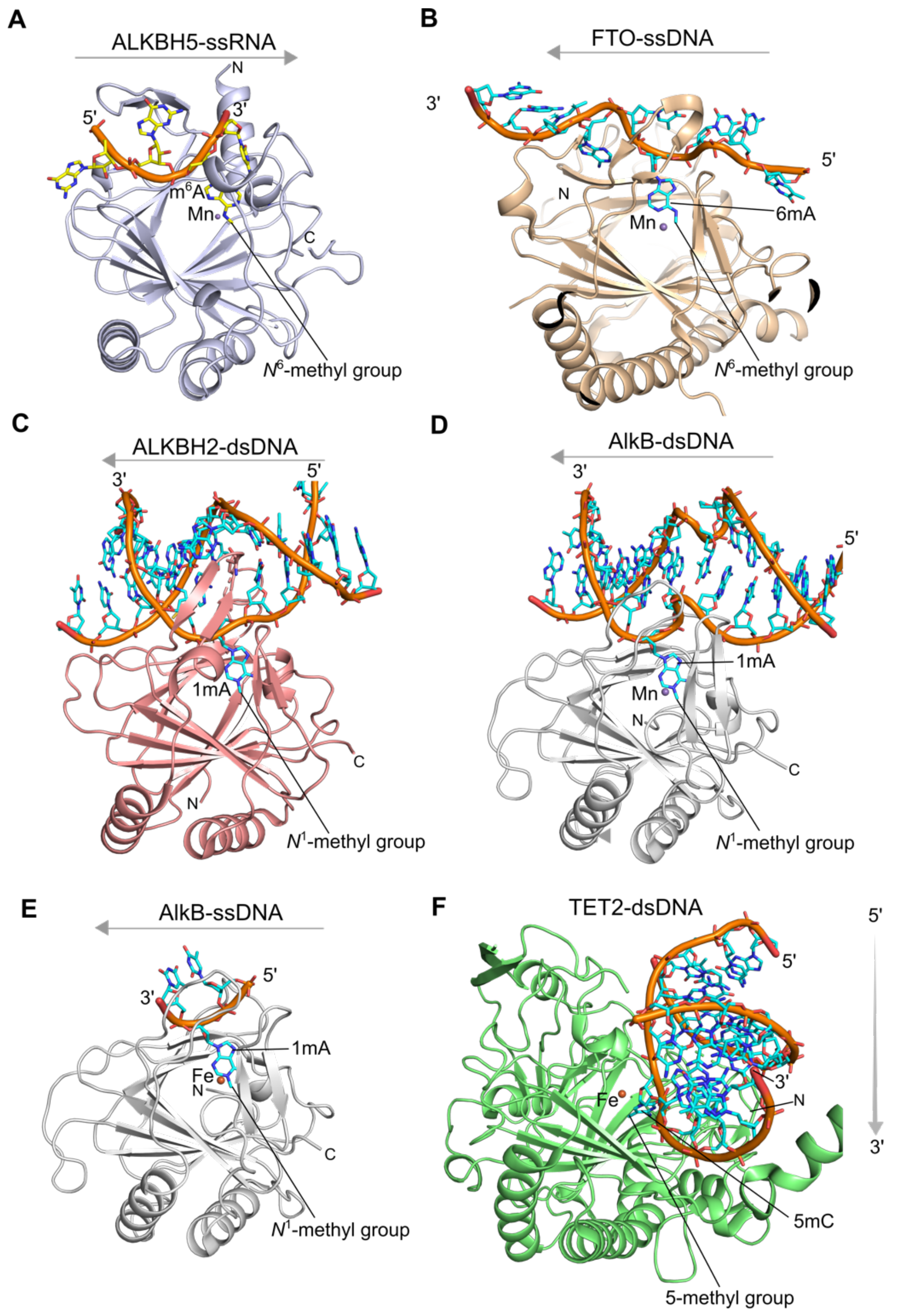

Figure S12. Comparison of nucleic acid substrate binding modes for the AlkB subfamily 2OG oxygenases. (A) ALKBH5 (light blue) (PDB ID 7WKV), (B) FTO (light brown) (PDB ID 5ZMD) (7), (C) ALKBH2 (salmon) (PDB ID 3BTY) (8), (D) AlkB (grey) (PDB ID 3BIE) in complex with dsDNA (8), (E) AlkB (grey) (PDB ID 2FD8) in complex with trinucleotide ssDNA (9), and (F) TET2 (PDB ID 4NM6) (10). RNA is in yellow sticks and DNA is in cyan sticks. Light grey arrows indicate the 5′ to 3′ direction of the nucleic acid strands through the active sites. Note: the direction of the dsDNA bound to TET2 is almost perpendicular to substrates bound to members of AlkB subfamily (views are from a similar orientation).

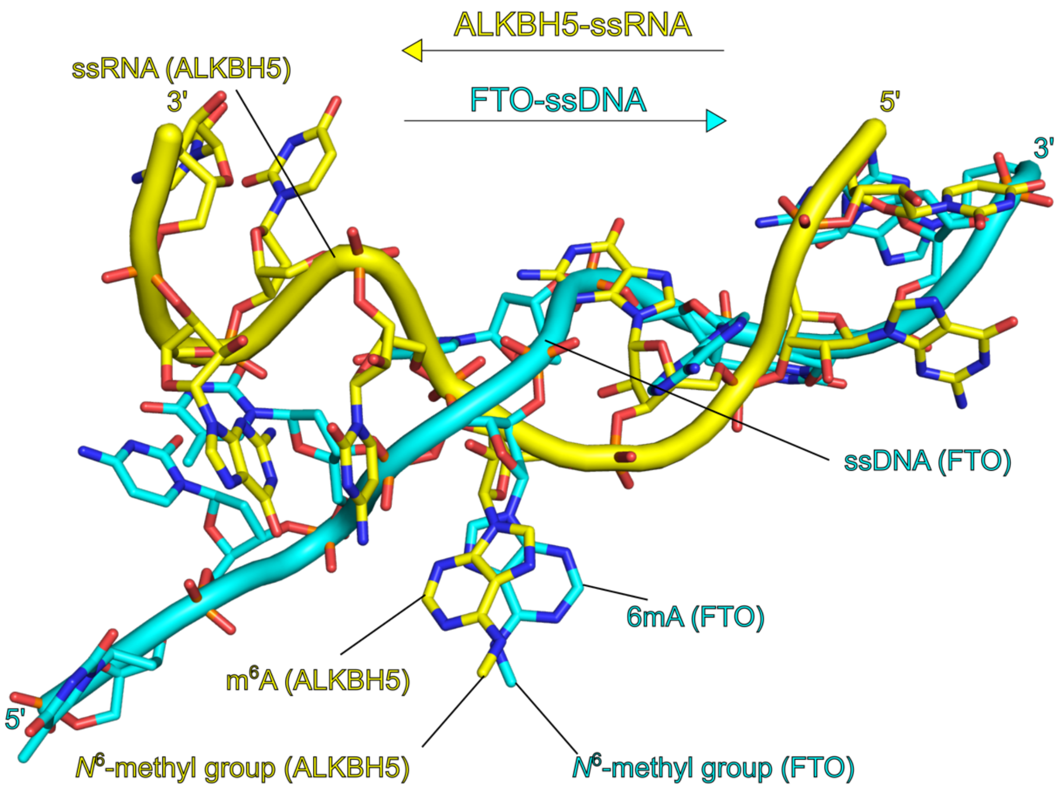

Figure S13. The opposing 5′ to 3′ directions through the active sites of oligonucleotide substrates when bound to ALKBH5$_{74\text{-}292}$ or an FTO variant. Overlay of the ssRNA (yellow ribbon and sticks) and ssDNA (cyan ribbon and sticks) from the superimposed structures of the ALKBH5$_{74\text{-}292}$-NOG-ssRNA (PDB ID 7WL0)) and the FTO-ssDNA (PDB ID 5ZMD) (7) complexes, respectively. Colors: ssRNA bound to ALKBH5, yellow, ssDNA bound to FTO, cyan; O, red; N, blue; P, orange. The m^{6}A and 6mA binding modes in ALKBH5$_{74\text{-}292}$ and the FTO variant, respectively, are flipped by ~180° relative to each other in the active site. Arrows indicate the 5′ to 3′ direction of the nucleic acid strands (ALKBH5, yellow; FTO, cyan).

Table S1. X-Ray data collection and refinement statistics.

	ALKBH5-RNA	ALKBH5-2OG-RNA	ALKBH5-NOG-RNA
**PDB ID**	**7V4G**	**7WKV**	**7WL0**
**Radiation Source**	Diamond Light Source I03	Diamond Light Source I24	Diamond Light Source I24
**Detector**	Eiger2 XE 16M	Pilatus3 6M	Pilatus3 6M
**X-ray Wavelength, Å**	0.9763	0.9999	0.9999
**Resolution Range†, Å**	41.46 - 2.10 (2.18 - 2.10)	41.94 - 2.10 (2.18 - 2.10)	51.55 – 2.50 (2.59 – 2.50)
**Space Group**	$P3_2$	$P3_2$	$P2_1$
**Unit Cell Dimensions**	a = 76.5 Å, b = 76.5 Å, c = 106.3 Å, α = 90°, β = 90°, γ = 120°	a = 78.4 Å, b = 78.4 Å, c = 106.7 Å, α = 90°, β = 90°, γ = 120°	a = 59.8 Å, b = 132.4 Å, c = 78.0 Å, α = 90°, β = 100.99°, γ = 90°
**Total No. of Reflections Observed†**	201030 (20409)	159355 (16053)	576964 (48279)
**No. of Unique Reflections†**	40605 (4132)	42760 (4300)	41144 (4103)
**Multiplicity†**	5.0 (4.9)	3.7 (3.7)	14.0 (11.8)
**Completeness†, %**	99.97 (99.93)	99.68 (99.07	99.92 (99.81)
**$CC_{1/2}$†**	0.987 (0.536)	0.935 (0.681)	0.981 (0.42)
**$I/\sigma(I)$†**	8.61 (1.40)	11.80 (1.00)	4.76 (1.17)
**Wilson B-Factor, Å²**	22.79	35.53	42.40
**R_{work}†**	0.1850 (0.2362)	0.1880 (0.3286)	0.1974 (0.3067)
**R_{free}†**	0.2236 (0.2646)	0.2274 (0.3741)	0.2402 (0.3784)
**RMS Deviation (Bonds/Angles)**	0.007 Å / 0.99°	0.004 Å / 0.71°	0.003 Å / 0.52°
**Ramachandran Outliers, %**	0.00	0.00	0.00
**Average B-Factor (All Atoms), Å²**	30.97	50.50	49.19
**Average B-Factor (Protein), Å²**	31.02	50.51	48.02
**Average B-Factor (RNA), Å²**	31.62	54.28	67.36
**Average B-Factor (Ligands), Å²**	26.47	44.63	44.80
**Average B-Factor (Solvent), Å²**	28.72	44.09	42.12
**No. of Non-H Atoms (macromolecules)**	5224	5336	9014
**No. of Non-H Atoms (Ligands)**	111	102	173
**No. of Non-H Atoms (Solvent)**	235	89	175

† Statistics for the highest-resolution shell are in parentheses.
R_{free} is based on 5.0 % of the reflections used in refinement.

Table S2. DNA primer sequences used for cloning and site-directed mutagenesis using transfer-PCR.

	Forward Primer (5′ to 3′)	Reverse Primer (5′ to 3′)
ALKBH5$_{74-292}$ wildtype	GAGAACCTGTACTTCCAATCCCA GCAGCTGCAGAAGGAGG	CTCCTTCTGCAGCTGCTGGG ATTGGAAGTACAGGTTCTC
ALKBH5$_{74-292}$ R148A	CAGCTGCAGAAGGCCGGGCCC GGCCA	TGGCCGGGCCCGGCCTTCT GCAGCTG
ALKBH5$_{74-292}$ R148E	CCAGCTGCAGAAGGAGGGGCC CGGCCAGG	CCGCTGAGCAATAACTAGC
ALKBH5$_{74-292}$ R130A	GACCGGGCCCCACTGGCCAACA AGTACTTCTTC	CCGCTGAGCAATAACTAGC
ALKBH5$_{74-292}$ R130E	GGACCGGGCCCCACTGGAGAA CAAGTACTTCTTCGG	CCGCTGAGCAATAACTAGC
ALKBH5$_{74-292}$ Y139A	TCTTCGGCGAAGGCGCCACTTA CGGCGCCC	CCGCTGAGCAATAACTAGC
ALKBH5$_{74-292}$ H204A	GCGGCTGCATCGTGTCTGCCGT GGACCC	CCGCTGAGCAATAACTAGC
ALKBH5$_{74-292}$ F234A	CGGCTGCAAGTTCCAGGCCAAG CCTATTCGGGTG	CCGCTGAGCAATAACTAGC
ALKBH5$_{74-292}$ F232A	CTGTGCTTCGGCTGCAAGGCCC AGTTCAAGCCTATTCG	CCGCTGAGCAATAACTAGC
ALKBH5$_{74-292}$ F232A/F234A	CTGTGCTTCGGCTGCAAGGCCC AGGCCAAGCCTATTCGGGTGTC	CCGCTGAGCAATAACTAGC
ALKBH5$_{74-292}$ Y139F	TTCGGCGAAGGCTTCACTTACG GCGCC	CCGCTGAGCAATAACTAGC
ALKBH5$_{74-292}$ K132E	CCCCACTGCGCAACGAGTACTT CTTCGGC	CCGCTGAGCAATAACTAGC
ALKBH5$_{74-292}$ K132R	CCCACTGCGCAACAGGTACTTC TTCGGCG	CCGCTGAGCAATAACTAGC

Table S3. 8-mer m^6A-containing single-stranded RNA substrate sequences**.**

No.	RNA sequence
1	5′-UGG(m^6A)CUGC-3′
2	5′-UGA(m^6A)CUGC-3′
3	5′-UGU(m^6A)CUGC-3′
4	5′-UGC(m^6A)CUGC-3′
5	5′-UGG(m^6A)GUGC-3′
6	5′-UGG(m^6A)AUGC-3′
7	5′-UGG(m^6A)UUGC-3′
8	5′-UGC(m^6A)AUGC-3′
9	5′-UUG(m^6A)CUGC-3′
10	5′-UAG(m^6A)CUGC-3′
11	5′-UCG(m^6A)CUGC-3′

SUPPLEMENTARY REFERENCES

1. Fu,Y., Jia,G., Pang,X., Wang,R.N., Wang,X., Li,C.J., Smemo,S., Dai,Q., Bailey,K.A., Nobrega,M.A., *et al.* (2013) FTO-mediated formation of N6-hydroxymethyladenosine and N6-formyladenosine in mammalian RNA. *Nat. Commun.*, **4**, 1798.
2. Aik,W., Scotti,J.S., Choi,H., Gong,L., Demetriades,M., Schofield,C.J. and McDonough,M.A. (2014) Structure of human RNA N6-methyladenine demethylase ALKBH5 provides insights into its mechanisms of nucleic acid recognition and demethylation. *Nucleic Acids Res.*, **42**, 4741–4754.
3. Xu,C., Liu,K., Tempel,W., Demetriades,M., Aik,W., Schofield,C.J. and Min,J. (2014) Structures of Human ALKBH5 Demethylase Reveal a Unique Binding Mode for Specific Single-stranded N6-Methyladenosine RNA Demethylation. *J. Biol. Chem.*, **289**, 17299–17311.
4. Feng,C., Liu,Y., Wang,G., Deng,Z., Zhang,Q., Wu,W., Tong,Y., Cheng,C. and Chen,Z. (2014) Crystal Structures of the Human RNA Demethylase Alkbh5 Reveal Basis for Substrate Recognition. *J. Biol. Chem.*, **289**, 11571–11583.
5. Wallace,A.C., Laskowski,R.A. and Thornton,J.M. (1995) LIGPLOT: a program to generate schematic diagrams of protein-ligand interactions. *Protein Eng. Des. Sel.*, **8**, 127–134.
6. Laskowski,R.A. and Swindells,M.B. (2011) LigPlot+: Multiple Ligand–Protein Interaction Diagrams for Drug Discovery. *J. Chem. Inf. Model.*, **51**, 2778–2786.
7. Zhang,X., Wei,L.-H., Wang,Y., Xiao,Y., Liu,J., Zhang,W., Yan,N., Amu,G., Tang,X., Zhang,L., *et al.* (2019) Structural insights into FTO's catalytic mechanism for the demethylation of multiple RNA substrates. *Proc. Natl. Acad. Sci.*, **116**, 2919–2924.
8. Yang,C.-G., Yi,C., Duguid,E.M., Sullivan,C.T., Jian,X., Rice,P.A. and He,C. (2008) Crystal structures of DNA/RNA repair enzymes AlkB and ABH2 bound to dsDNA. *Nature*, **452**, 961–965.
9. Yu,B., Edstrom,W.C., Benach,J., Hamuro,Y., Weber,P.C., Gibney,B.R. and Hunt,J.F. (2006) Crystal structures of catalytic complexes of the oxidative DNA/RNA repair enzyme AlkB. *Nature*, **439**, 879–884.
10. Hu,L., Li,Z., Cheng,J., Rao,Q., Gong,W., Liu,M., Shi,Y.G., Zhu,J., Wang,P. and Xu,Y. (2013) Crystal Structure of TET2-DNA Complex: Insight into TET-Mediated 5mC Oxidation. *Cell*, **155**, 1545–1555.
